# Comparison of Methods To Collect Fecal Samples for Microbiome Studies Using Whole-Genome Shotgun Metagenomic Sequencing

**DOI:** 10.1128/mSphere.00827-19

**Published:** 2020-02-26

**Authors:** Doratha A. Byrd, Rashmi Sinha, Kristi L. Hoffman, Jun Chen, Xing Hua, Jianxin Shi, Nicholas Chia, Joseph Petrosino, Emily Vogtmann

**Affiliations:** a Metabolic Epidemiology Branch, Division of Cancer Epidemiology & Genetics, National Cancer Institute, National Institutes of Health, Bethesda, Maryland, USA; b Biostatistics Branch, Division of Cancer Epidemiology & Genetics, National Cancer Institute, Bethesda, Maryland, USA; c Alkek Center for Metagenomics and Microbiome Research, Department of Molecular Virology and Microbiology, Baylor College of Medicine, Houston, Texas, USA; d Microbiome Program, Center for Individualized Medicine, Mayo Clinic, Rochester, Minnesota, USA; e Health Sciences Research, Mayo Clinic, Rochester, Minnesota, USA; f Department of Surgery, Mayo Clinic, Rochester, Minnesota, USA; g Biomedical Engineering and Physiology, Mayo Clinic, Rochester, Minnesota, USA; h Human Genome Sequencing Center, Baylor College of Medicine, Houston, Texas, USA; University of Michigan—Ann Arbor

**Keywords:** whole-genome shotgun sequencing, microbiome, fecal sample collection method, FOBT cards, FIT tubes

## Abstract

Few previous studies have assessed stability and “gold-standard” concordance of fecal sample collection methods for whole-genome shotgun metagenomic sequencing (WGSS), an increasingly popular method for studying the gut microbiome. We used WGSS data to investigate ambient temperature stability and putative gold-standard concordance of microbial profiles in fecal samples collected and stored using fecal occult blood test (FOBT) cards, fecal immunochemical test (FIT) tubes, 95% ethanol, or RNAlater. Among 15 Mayo Clinic employees, for each collection method, we calculated intraclass correlation coefficients (ICCs) to estimate stability of fecal microbial profiles after storage for 4 days at ambient temperature and concordance with immediately frozen, no-solution samples (i.e., the putative gold standard). ICCs were estimated for multiple metrics, including relative abundances of select phyla, species, KEGG k-genes (representing any coding sequence that had >70% identity and >70% query coverage with respect to a known KEGG ortholog), KEGG modules, and KEGG pathways; species and k-gene alpha diversity; and Bray-Curtis and Jaccard species beta diversity. ICCs for microbial profile stability were excellent (≥90%) for fecal samples collected via most of the collection methods, except those preserved in 95% ethanol. Concordance with the immediately frozen, no-solution samples varied for all collection methods, but the number of observed species and the beta diversity metrics tended to have higher concordance than other metrics. Our findings, taken together with previous studies and feasibility considerations, indicated that FOBT cards, FIT tubes, and RNAlater are acceptable choices for fecal sample collection methods in future WGSS studies.

**IMPORTANCE** A major direction for future microbiome research is implementation of fecal sample collections in large-scale, prospective epidemiologic studies. Studying microbiome-disease associations likely requires microbial data to be pooled from multiple studies. Our findings suggest collection methods that are most optimal to be used standardly across future WGSS microbiome studies.

## Abstract (precis)

A major direction for future microbiome research is implementation of fecal sample collections in large-scale, prospective epidemiologic studies. Studying microbiome-disease associations likely requires microbial data to be pooled from multiple studies. Our findings suggest collection methods that are most optimal to be used standardly across future WGSS microbiome studies.

## INTRODUCTION

Growing evidence suggests that the gut microbiome is involved in the etiology of multiple chronic diseases, including certain cancers (1–6). However, vast gaps in knowledge exist regarding microbiome-disease associations, exacerbated by inconsistent findings across studies which may be due, in part, to differences in fecal sample collection and storage, DNA extraction and amplification, bioinformatic procedures, and other factors (7, 8).

A major direction for future microbiome research is implementation of fecal sample collections in large-scale, prospective epidemiologic studies. Evidence from 16S rRNA gene amplicon sequencing studies suggests that the chosen fecal collection method impacts multiple estimates of microbial composition (9–12); thus, development of standard fecal collection protocols is required, especially for pooling of microbial data across studies. Ideal fecal sample collection methods preserve the original microbial signature of the fecal sample, stabilize microbial DNA, and prevent bacterial growth during room-temperature storage for multiple days, which is typical under field conditions (10, 13–16).

While 16S rRNA gene amplicon sequencing is commonly used to characterize bacterial profiles, researchers are increasingly using whole-genome shotgun metagenomic sequencing (WGSS) to sequence the genome of all microorganisms in biological samples. Compared to 16S rRNA gene studies, WGSS provides high-resolution profiles of bacteria down to the species or strain level and can estimate functional potential of microbes using gene/pathway content information, which may be particularly important for understanding the role of the microbiota in human health (17–19).

Only one small study (*n* = 8) (20) investigated stability and “gold-standard” concordance of WGSS fecal samples, collected using 100% ethanol or RNAlater. That study did not consider other common, established methods for fecal sample collection such as fecal occult blood test (FOBT) cards and fecal immunochemical tests (FIT), which were recently shown to be reproducible, stable, and relatively concordant with the “gold-standard” collection method in 16S rRNA gene studies (10–12). FOBT cards and FIT tests are used widely in colorectal cancer screening, providing opportunities for establishing prospective cohorts using discarded tests. We used WGSS to comprehensively characterize the microbiota of fecal samples in a subset (*n* = 15) of individuals from a previous 16S rRNA gene sequencing study, the Mayo 2 study (11). Here, we report an investigation of the stability and “gold-standard” concordance of the WGSS profiles in fecal samples collected using four methods: FOBT cards, FIT tubes, 95% ethanol, and RNAlater.

## RESULTS

Participants predominantly were non-Hispanic white (86.7%), had a bowel movement at least once per day (80.0%), and were nonsmokers (100%). About half were female (53.3%). On average, they were 37.5 years old, with ages ranging from 22 to 53 (see Table S1 in the supplemental material).

10.1128/mSphere.00827-19.3 TABLE S1 Characteristics of study participants in the Mayo 2 study, Rochester, MN, 2014 (*n* = 15). Download Table S1, DOCX file, 0.01 MB. Copyright © 2020 Byrd et al. 2020 Byrd et al. This content is distributed under the terms of the Creative Commons Attribution 4.0 International license.

### Alpha diversity by collection method.

**FIG 1:**
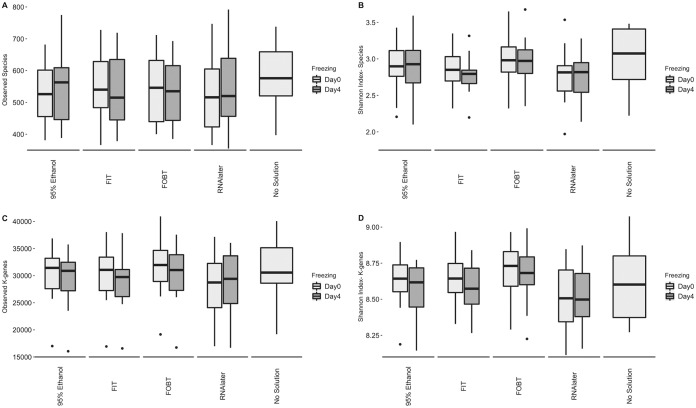
Box plots for observed species (A), Shannon index estimates based on species (B), observed k-genes (C), and Shannon index estimates based on k-genes (D) by fecal sample collection method on day-0 and day-4 of freezing in fecal samples from the Mayo 2 study, Rochester, MN, 2014 (*n* = 15). Abbreviations: FIT, fecal immunochemical test tubes; FOBT, fecal occult blood test cards.

As shown in Fig. 1, the number of observed species and the Shannon index values for species were generally highest in samples without solution, frozen on day-0 (the “gold standard”). The number of observed k-genes (representing any coding sequence that had >70% identity and >70% query coverage with respect to a known KEGG ortholog) and the Shannon index values for k-genes tended to be higher on day-0 of freezing, except in RNAlater samples, and were highest, on average, in FOBT card samples for both metrics. After adjusting for freezing time in linear mixed-effects models (Table S2), compared to the “gold standard,” averages of 46, 31, 41, and 43 fewer species were detected in samples collected via 95% ethanol, FIT tubes, FOBT cards, and RNAlater, respectively (*P = *0.001, 0.02, 0.002, and 0.001, respectively). In RNAlater samples, the Shannon index values for species and the number of observed k-genes were lower than were seen with the “gold standard,” and the Shannon index value for k-genes was highest in FOBT card samples compared to all other samples.

10.1128/mSphere.00827-19.4 TABLE S2 Adjusted means for observed species/k-genes and the Shannon index value for species/k-genes by fecal sample collection method in fecal samples from the Mayo 2 study, Rochester, MN, 2014 (*n* = 15). Abbreviations: FIT, fecal immunochemical test tubes; FOBT, fecal occult blood test cards. Download Table S2, DOCX file, 0.01 MB. Copyright © 2020 Byrd et al. 2020 Byrd et al. This content is distributed under the terms of the Creative Commons Attribution 4.0 International license.

### Percent variability explained by subject, collection method, and storage time.

Based on the Bray-Curtis and Jaccard distance matrices, the percentage of variability in species and k-genes was primarily explained by subject and minimally by collection method and storage time (see Fig. S1 in the supplemental material; see also Table S3). For example, based on the Bray-Curtis distance matrix, interindividual variability explained 71% and 68% of variability in species and k-genes, respectively; whereas the collection method explained 9% and 10%, respectively; and storage time explained <1% of variability for both metrics.

10.1128/mSphere.00827-19.1 FIG S1 Percent variability explained by subject, sample collection type, and day of freezing using a distance-based coefficient of determination (*R*^2^) for beta diversity estimates from Bray-Curtis and Jaccard distance matrices for (A) species and (B) k-genes in the Mayo 2 study, Rochester, MN, 2014 (*n* = 15). Download FIG S1, TIF file, 2.1 MB. Copyright © 2020 Byrd et al. 2020 Byrd et al. This content is distributed under the terms of the Creative Commons Attribution 4.0 International license.

10.1128/mSphere.00827-19.5 TABLE S3 Percent variability explained by subject, sample collection type, and day of freezing using a distance-based coefficient of determination (*R*^2^) for beta diversity estimates from Bray-Curtis and Jaccard distance matrices for species and k-genes in the Mayo 2 study, Rochester, MN, 2014 (*n* = 15). Download Table S3, DOCX file, 0.01 MB. Copyright © 2020 Byrd et al. 2020 Byrd et al. This content is distributed under the terms of the Creative Commons Attribution 4.0 International license.

### Relative abundance comparisons.

**FIG 2:**
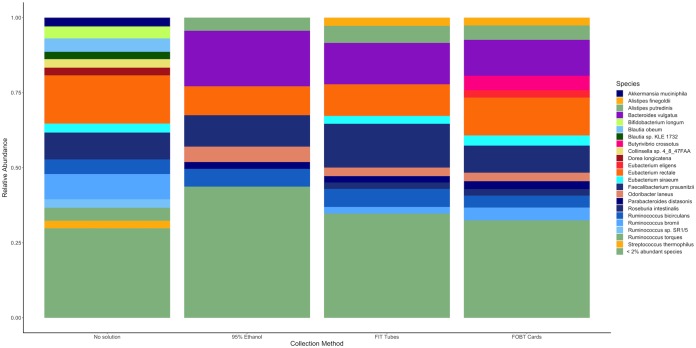
Relative abundance of bacterial species present in at least 50% of fecal samples with relative abundance of ≥2% in fecal samples frozen day-0 in the Mayo 2 study, Rochester, MN, 2014 (*n* = 15). Abbreviations: FIT Tubes, fecal immunochemical test tubes; FOBT Cards, fecal occult blood test cards.

As shown in Fig. 2 (and in Fig. S2, by sample), samples collected without solution and frozen day-0 had relative abundances that were ≥2% of those calculated for certain species, such as Akkermansia muciniphila, Bifidobacterium longum, Blautia obeum, *Blautia* sp. *KLE 1732*, *Collinsella* sp. *4_8_47FAA*, and Streptococcus thermophilus, that were not detected at abundances of ≥2% by the other collection methods similarly frozen day-0. For the other collection methods, the general distributions of relative abundances of each species were fairly inconsistent, but the differences between those methods were smaller than those seen with the samples without solution.

10.1128/mSphere.00827-19.2 FIG S2 Relative abundance of bacterial species present in at least 50% of fecal samples with relative abundance of >2% in fecal samples frozen day 0 by individual samples in the Mayo 2 study, Rochester, MN, 2014 (*n* = 15). Abbreviations: FIT, fecal immunochemical test tubes; FOBT, fecal occult blood test cards. Download FIG S2, TIF file, 2.7 MB. Copyright © 2020 Byrd et al. 2020 Byrd et al. This content is distributed under the terms of the Creative Commons Attribution 4.0 International license.

### Stability.

**FIG 3:**
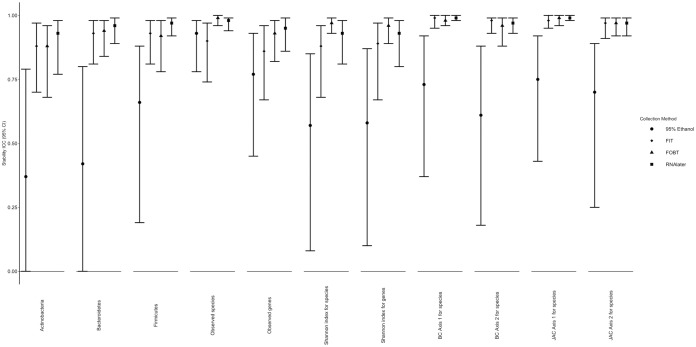
ICCs for stability of microbial diversity metrics by fecal sample collection method comparing fecal samples frozen on day-0 to those frozen on day-4 in the Mayo 2 study, Rochester, MN, 2014 (*n* = 15); phylum relative abundances were square root transformed prior to calculating ICCs. Abbreviations: BC, Bray-Curtis principal coordinate; FIT, fecal immunochemical test tubes; FOBT, fecal occult blood test cards; ICC, intraclass correlation coefficient; JAC, Jaccard principal coordinate.

Intraclass correlation coefficients (ICCs) comparing fecal samples frozen day-4 to those frozen day-0 by collection method are shown in Fig. 3 (exact ICCs and 95% confidence intervals [CIs] are listed in Table S4). Stability ICCs for the most dominant phyla, alpha diversity of species and k-genes, and the first two principal coordinates of Bray-Curtis and Jaccard distance matrices for species were mostly ≥90% in samples collected using the FIT, FOBT, and RNAlater collection methods. Stability ICCs were generally lower and more variable in samples collected using 95% ethanol.

10.1128/mSphere.00827-19.6 TABLE S4 ICCs for stability of microbial diversity metrics by fecal sample collection method comparing fecal samples frozen on day-0 to those frozen on day-4 in the Mayo 2 study, Rochester, MN, 2014 (*n* = 15). Abbreviations: BC, Bray-Curtis principal coordinate; FIT, fecal immunochemical test tubes; FOBT, fecal occult blood test cards; ICC, intraclass correlation coefficient; JAC, Jaccard principal coordinate. Download Table S4, DOCX file, 0.01 MB. Copyright © 2020 Byrd et al. 2020 Byrd et al. This content is distributed under the terms of the Creative Commons Attribution 4.0 International license.

**FIG 4:**
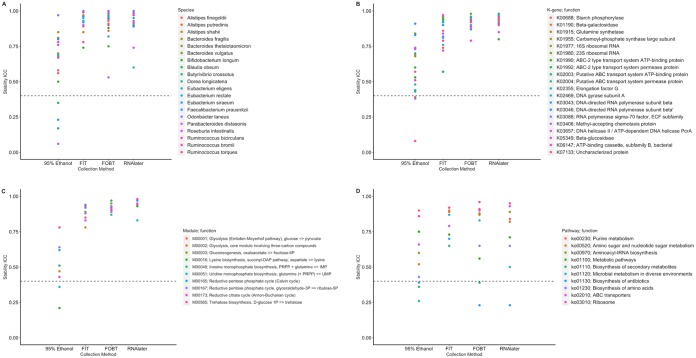
ICCs for stability of the relative abundance of the most abundant species (A), k-genes (B), modules (C), and pathways (D) by fecal sample collection method comparing fecal samples frozen on day-0 to those frozen on day-4 in the Mayo 2 study, Rochester, MN, 2014 (*n* = 15); species, k-gene, module, and pathway relative abundances were square root transformed prior to calculating ICCs. Abbreviations: FIT, fecal immunochemical test tubes; FOBT, fecal occult blood test cards; ICC, intraclass correlation coefficient; DAP, diaminopimelate; PRPP, 5-phosphoribosyl-1-pyrophosphate.

Stability ICCs for the most abundant species, k-genes, modules, and pathways are shown in Fig. 4 (exact ICCs and 95% confidence intervals are listed in Table S5) for each collection method. Similarly to the diversity metrics, ICCs for samples collected using FOBT, FIT, and RNAlater were generally higher than those for 95% ethanol samples. In the 95% ethanol samples, 4 of 20 species, 3 of 20 k-genes, 2 of 10 modules, and 3 of 10 pathways had ICCs representing poor (<40%) stability. There were no substantial differences in the stability ICCs calculated based on the centered log ratio-transformed and untransformed relative abundances (data not shown), except that the ICCs for centered log ratio-transformed pathways were generally lower.

10.1128/mSphere.00827-19.7 TABLE S5 ICCs for stability of the square root-transformed relative abundance of the most abundant species, k-genes, modules, and pathways by fecal sample collection method comparing fecal samples frozen on day-0 to those frozen on day-4 in the Mayo 2 study, Rochester, MN, 2014 (*n* = 15). Abbreviations: FIT, fecal immunochemical test tubes; FOBT, fecal occult blood test cards; ICC, intraclass correlation coefficient. Download Table S5, DOCX file, 0.02 MB. Copyright © 2020 Byrd et al. 2020 Byrd et al. This content is distributed under the terms of the Creative Commons Attribution 4.0 International license.

### Concordance.

**FIG 5:**
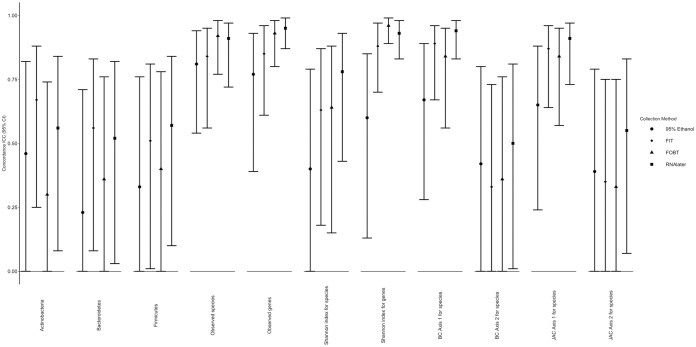
ICCs for concordance of microbiome diversity metrics of each fecal sample collection method compared to the putative “gold-standard” (samples with no solution, frozen immediately) in fecal samples from the Mayo 2 study, Rochester, MN, 2014 (*n* = 15); phylum relative abundances were square root transformed prior to calculating ICCs. Abbreviations: BC, Bray-Curtis principal-coordinate vector; FIT, fecal immunochemical test tubes; FOBT, fecal occult blood test cards; ICC, intraclass correlation coefficient; JAC, Jaccard principal-coordinate vector.

ICCs comparing samples without solution frozen on day-0 (i.e., the putative “gold standard”) to samples collected using the other methods (also frozen day-0) are shown in Fig. 5 (exact ICCs, the corresponding calculated Spearman correlations [SCCs], and their 95% CIs are listed in Table S6). Generally, the confidence intervals were wide, indicating high variability in “gold-standard” concordance. ICCs were lowest for samples collected using 95% ethanol and FOBT cards for relative abundance of *Actinobacteria* (0.46 and 0.30, respectively), *Bacteroidetes* (0.23 and 0.36, respectively), and *Firmicutes* (0.33 and 0.40, respectively); whereas for FIT tubes and RNAlater, the corresponding ICCs were >0.50. ICCs for observed species were ≥75% for all collection methods. For the rest of the alpha diversity metrics, ICCs were generally highest in FOBT and RNAlater samples and lowest in 95% ethanol samples. ICCs were ≥75% for the first principal-coordinate vectors of both species Bray-Curtis and Jaccard distances for all collection methods, except for 95% ethanol samples with slightly lower ICCs, but for all collection methods, ICCs were substantially lower for the second principal-coordinate vector of both distance matrices (i.e., all ICCs were <40%, except in RNAlater samples). Spearman correlations (SCCs) (Table S6) comparing the rank order of each metagenomic diversity metric to those for the “gold standard” varied between being slightly higher or lower than the ICCs.

10.1128/mSphere.00827-19.8 TABLE S6 ICCs and Spearman correlation coefficients for concordance of microbiome diversity metrics of each fecal sample collection method compared to the putative “gold-standard” (samples with no solution, frozen immediately) in fecal samples from the Mayo 2 study, Rochester, MN, 2014 (*n* = 15). Abbreviations: BC, Bray-Curtis principal coordinate; FIT, fecal immunochemical test tubes; FOBT, fecal occult blood test cards; ICC, intraclass correlation coefficient; JAC, Jaccard principal coordinate; Rs, Spearman correlation coefficient. Download Table S6, DOCX file, 0.01 MB. Copyright © 2020 Byrd et al. 2020 Byrd et al. This content is distributed under the terms of the Creative Commons Attribution 4.0 International license.

**FIG 6:**
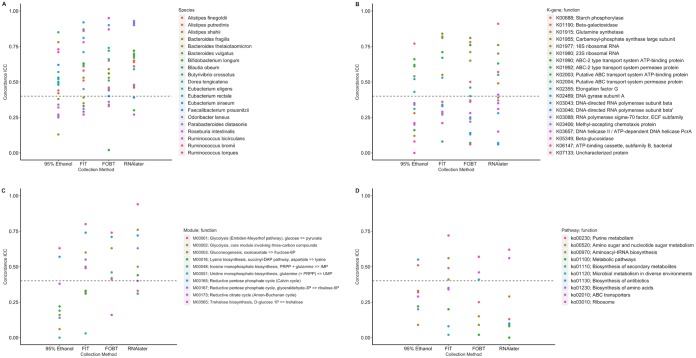
ICCs for concordance of the relative abundance of the most abundant species (A), k-genes (B), modules (C), and pathways (D) for each fecal sample collection method the putative “gold-standard” (samples with no solution, frozen immediately) in the Mayo 2 study, Rochester, MN, 2014 (*n* = 15); species, k-gene, module, and pathway relative abundances were square root transformed prior to calculating ICCs. Abbreviations: FIT, fecal immunochemical test tubes; FOBT, fecal occult blood test cards; ICC, intraclass correlation coefficient.

Concordance ICCs for the most abundant species, k-genes, modules, and pathways are shown in Fig. 6 and listed in Table S7 (along with corresponding SCCs) for each fecal sample collection method. Seven, six, five, and three of the 20 most abundant species had poor concordance ICCs in 95% ethanol, FIT tubes, FOBT cards, and RNAlater samples, respectively. Concordance ICCs for k-genes, modules, and pathways varied greatly (e.g., range of concordance ICCs for k-genes in FOBT and RNAlater samples, 0.06 to 0.81 and 0.06 to 0.91, respectively), and occurrences of ICCs that were <40% were frequent across all methods; however, the 95% ethanol samples tended to have the highest proportion of metrics with ICC estimates of <40%. Concordance ICCs calculated based on the centered log ratio-transformed and untransformed relative abundances varied between being higher or lower than those calculated based on the square root abundances, except that the ICCs for centered log ratio-transformed species tended to be much higher (data not shown). Finally, concordance ICCs for the microbiome metrics for each pairwise comparison of the collection methods varied greatly but were generally >40%. ICCs were highest comparing FOBT cards and RNAlater (Table S8).

10.1128/mSphere.00827-19.9 TABLE S7 ICCs and Spearman correlation coefficients for concordance of the square root-transformed relative abundance of the most abundant species, k-genes, modules, and pathways of each fecal sample collection method compared to the putative “gold-standard” (samples with no solution, frozen immediately) in fecal samples from the Mayo 2 study, Rochester, MN, 2014 (*n* = 15). Abbreviations: BC, Bray-Curtis principal coordinate; FIT, fecal immunochemical test tubes; FOBT, fecal occult blood test cards; ICC, intraclass correlation coefficient; JAC, Jaccard principal coordinate; Rs, Spearman correlation coefficient. Download Table S7, PDF file, 0.1 MB. Copyright © 2020 Byrd et al. 2020 Byrd et al. This content is distributed under the terms of the Creative Commons Attribution 4.0 International license.

10.1128/mSphere.00827-19.10 TABLE S8 ICCs for concordance of microbiome diversity metrics of each fecal sample collection method comparison combination in fecal samples from the Mayo 2 study, Rochester, MN, 2014 (*n* = 15). Abbreviations: BC, Bray-Curtis principal coordinate; FIT, fecal immunochemical test tubes; FOBT, fecal occult blood test cards; ICC, intraclass correlation coefficient; JAC, Jaccard principal coordinates. Download Table S8, DOCX file, 0.02 MB. Copyright © 2020 Byrd et al. 2020 Byrd et al. This content is distributed under the terms of the Creative Commons Attribution 4.0 International license.

## DISCUSSION

In this study of the stability and “gold-standard” concordance of WGSS profiles of fecal samples collected using FOBT cards, FIT tubes, RNAlater, and 95% ethanol, our results support the conclusions that (i) the variation in WGSS profiles is primarily explained by interindividual differences and substantially less so by collection method or days of storage at ambient temperature; (ii) the distribution of species-level relative abundances differ across collection methods, particularly so for samples collected, frozen, and thawed without solution; (iii) all collection methods, except for 95% ethanol, adequately preserved WGSS bacterial profiles after 4 days at ambient temperature; and (iv) compared to immediate freezing of no-solution samples, all collection methods tended to be most concordant for the observed species and the first principal-coordinate vectors for Bray-Curtis or Jaccard species distances.

The stability and “gold-standard” concordance of microbial profiles of fecal samples collected via the different methods were most extensively characterized using 16S rRNA gene sequencing (10–12, 21–25). Only one WGSS study, by Franzosa et al. (20), previously investigated the short-term stability and “gold-standard” concordance of fecal samples collected using RNAlater or 100% ethanol. After 2 days of storage at room temperature, they found that both methods preserved metagenomic profiles of fecal samples (similarly to our RNAlater findings) and that the results seen with both methods were comparable to those seen with the method employing immediately frozen samples without solution. Previously, Vogtmann et al. investigated the stability and accuracy of sequencing of 16S rRNA gene amplicon bacterial profiles of fecal samples collected via FOBT cards, FIT tubes, 95% ethanol, and RNAlater in the larger Mayo 2 study (*n* = 52) in which this study was nested (11). In analogy to our findings, they found that interindividual differences explained most variability in bacterial profiles and that, for the microbiome diversity metrics (comprising three phyla, alpha diversity metrics, and beta diversity metrics), the method employing 95% ethanol was generally the least stable of all the fecal sample collection methods.

While 95% ethanol was the only collection method seemingly unsuitable for storage of fecal samples at ambient temperature, no collection method was more suitably concordant with the “gold standard” than any other. Due to the nature of storage without solution (e.g., in the absence of DNA stabilizing or antimicrobial agents), growth of certain taxa is highly plausible (26), especially during freezing or thawing performed for extraction and sequencing or under typical field conditions where participants collect their own fecal samples at home and ship them to laboratories (10, 14). Therefore, though the use of immediately frozen samples without solution is considered the “gold standard” in the field (8, 23, 27–29), it is difficult to ascertain whether the results seen with the no-solution samples in this study could be confirmed as representative of the “truth”—a feat that would truly be possible only with immediate DNA extraction after defecation. Of concern, we observed greater relative abundances of certain species in no-solution samples than were seen with the other collection methods, similarly to an occurrence in a study by Bahl et al., which found that, compared to immediately extracted samples, samples that were frozen without solution differed in their *Firmicutes*-to-*Bacteroidetes* ratio (30). Since the relative abundance estimates were highly interrelated due to their compositionality, if bacterial growth occurred in the no-solution samples, our concordance estimates for all relative abundances would most likely be lower than the truth. This may be further evidenced by the higher concordance ICCs that we observed for centered log ratio-transformed species relative abundance (compared to square root-transformed abundance), which incorporate methods developed to account for relative abundance interrelatedness (31). Thus, taken together, our “concordance” findings should be interpreted with caution.

This study had several strengths. This was the first study to use WGSS to estimate the stability and concordance of bacteria in samples collected using four different methods. These collection methods were also shown to be generally stable and “gold-standard” concordant in 16S rRNA gene sequencing analyses of bacteria (10–12); most of these methods can be used for other -omics technologies, except for RNAlater, which cannot be used for metabolomics (21, 32); and FOBT cards and FIT tubes are cost-effective options that are increasingly used for colorectal cancer screening, opening opportunities for establishing prospective cohorts within screening populations. Furthermore, each collection method has its own advantages pertaining to stabilizing DNA, preventing bacterial growth, and preserving microbial profiles that are as close to those of the original sample as possible. For example, FIT tubes stabilize DNA via the use of antimicrobial agents (e.g., sodium azide) (8, 24); 95% to 99% ethanol has antimicrobial properties (8); and RNAlater acts as both a DNA stabilizer and an RNA stabilizer by preventing degradation by nucleases (33).

This study also had several limitations. This study was conducted in a mostly white, healthy population, which may limit generalizability with respect to populations with different environmental exposures; however, we previously found, using 16S rRNA gene amplicon sequencing data, that the levels of stability and “gold-standard” concordance of each fecal sample collection method were similar between populations in the United States and in Bangladesh (11, 12). Furthermore, in this study, stability was assessed only over the course of 4 days at ambient temperature, which may not necessarily directly reflect shipping conditions (which, e.g., might involve longer shipping times or storage in higher temperatures) and also does not necessarily reflect storage in freezers over long periods of time. Voigt et al. investigated long-term stability of WGSS profiles in fecal samples stored in RNAlater (*n* = 7) (34) and found that RNAlater preserved the microbiota composition well for up to 2 years of storage at –80°C. These findings warrant future studies of long-term stability using the collection methods described here.

### Conclusion.

These findings, taken together with previous 16S rRNA gene and other -omic stability and concordance studies, indicate that FOBT cards, FIT tubes, and RNAlater may be appropriate choices to collect fecal samples for WGSS in future studies, as these options are stable after storage at ambient temperature. Given the large gaps in knowledge pertaining to the role of the gut microbiome in the etiology of health and disease, ideally, one method should be used as the standard across studies so that WGSS data may be pooled to improve power to detect bacterial species/gene-level exposure/disease associations. Future studies should further investigate the long-term (over the course of years) stability of these collection methods in WGSS studies and should continue to explore the stability and concordance of each fecal collection method for other -omics technologies.

## MATERIALS AND METHODS

### Study population.

The Mayo 2 study was described previously (11). Briefly, among Mayo Clinic employees in Rochester, MN, 52 healthy volunteers were recruited and 15 participants from that group were randomly selected for this study. Eligible individuals were ≥18 years old, had not used antibiotics or probiotics within the 2 weeks prior, had no history of pelvic radiation, and were not receiving concurrent chemotherapy treatment. All participants gave informed consent, and the study was approved by the Mayo Clinic Studies Institutional Review Board.

### Data collection.

Each participant was provided an Exakt Pak canister (Inmark Packaging, Austell, GA) to collect feces. After defecation, the sample was delivered to the laboratory for immediate processing by a study coordinator. Diet and medical history were assessed with abbreviated questionnaires.

For each participant, fecal specimens were manually mixed using a spatula, and aliquots of samples obtained using the four different collection methods plus one sample without solution were generated in random order. For each participant, approximately 1 to 2 g of feces was placed in a Sarstedt feces tube (Numbrecht, Germany) without solution (4 aliquots), with 2.5 ml of RNAlater stabilization solution (4 aliquots), or with 2.5 ml of 95% ethanol (Sigma-Aldrich, St. Louis, MO; 4 aliquots). Four triple-slide Hemoccult II Elite Dispensapak Plus for FOBT cards (Beckman Coulter, Brea, CA) were smeared thinly with feces, and the flap was closed per manufacturer instructions. Four FIT tube aliquots (Polymedco, Inc., Cortlandt Manor, NY) were created by dipping the probe into the fecal specimen, shaking the tube, and transferring the fluid from one FIT tube into two cryovials.

All replicates of the no-solution samples and half of the 95% ethanol, RNAlater, FOBT card, and FIT tube replicates were frozen immediately (−80°C; day-0). To simulate testing for occult blood in colorectal cancer screening, the FOBT cards were developed with 2 drops of Hemoccult Sensa Developer (Beckman Coulter) applied to the guaiac paper on the back of the card immediately prior to freezing. The remaining half of each sample was stored at ambient temperature for 4 days and then frozen at −80°C (day-4). For each participant, one immediately frozen no-solution sample and two replicates frozen on day-0 and day-4 for all other collection methods were submitted for WGSS (*n* = 135 samples plus 14 duplicates).

### DNA extraction.

Fecal samples were submitted for WGSS in either solid or homogenized form or smeared onto FOBT cards. Homogenized stool samples (FIT tube, 95% ethanol, and RNAlater samples) were subjected to thorough vortex mixing, and 150 μl was aliquoted for DNA extraction. For solid stool (samples without solution), approximately 50 mg of sample was retrieved using a sterile spatula. For FOBT card smears, a squared area containing the majority of the stool was cut from the card. Then, all samples were transferred into a well on an extraction deep-well plate.

DNA extraction was conducted using a PowerMag soil DNA isolation kit (Mo Bio Laboratories, catalog no.27000-4) on a Hamilton STARlet automated liquid handler platform following the manufacturer’s instructions. DNA concentrations were estimated using the PicoGreen assay.

### Metagenomic WGS.

DNA (100 ng) was sheared into fragments of approximately 300 to 400 bp in a Covaris E210 system (Covaris, Inc., Woburn, MA) (96-well format) followed by purification of the fragmented DNA by the use of AMPure XP beads. DNA end repair, 3′ adenylation, ligation to Illumina multiplexing PE adaptors, and ligation-mediated PCR (LM-PCR) were all completed using automated processes. Kapa HiFi polymerase (Kapa Biosystems Inc.) was used for PCR amplification (8 cycles), which is known to amplify high-GC-rich and low-AT-rich regions at greater efficiency. A Fragment Analyzer electrophoresis system (Advanced Analytical Technologies, Inc.) was used for library quantification and size estimation. Prepared libraries (consisting of fragments that were, on average, 373 bp in size, including adapter and barcode) were then pooled in equimolar amounts to reach a final concentration of 20 nM and an average of 11 samples per pool.

Library templates were prepared for sequencing using Illumina’s cBot cluster generation system with TruSeq PE cluster generation kits. Briefly, the library templates was denatured with sodium hydroxide and diluted to 7 pM in hybridization buffer in order to achieve an average load density of 819,000 clusters/mm^2^. Each library pool was loaded in a single lane of a HiSeq 2000 flow cell spiked with a 1% phiX control library for run quality control. The sample then underwent bridge amplification to form clonal clusters, followed by hybridization with the sequencing primer. Sequencing runs were performed in paired-end mode using an Illumina HiSeq 2000 platform. Using TruSeq SBS kits, sequencing-by-synthesis reactions were extended for 101 cycles from each end, with an additional 10 cycles for the index read. After sequencing, .bcl files were processed through Illumina’s analysis software (bcl2fastq), which demultiplexes pooled samples and generates sequence reads and base-call confidence values (qualities). The average raw sequence yield per sample was 4.1 gigabases. One sample failed sequencing (an FOBT card sample frozen day-4).

### Metagenomic analysis.

Raw sequences were quality filtered and trimmed using a pipeline developed at the Baylor College of Medicine that employs a number of publicly available tools such as Casava v1.8.3 (Illumina) for the generation of fastqs and Trim Galore, PRINSEQ, and cutadapt for adapter and quality trimming. Bowtie2 v2.2.1 was used to remove reads that aligned to the hg38 human reference genome, and all remaining reads were mapped to a custom database that included all bacterial and archaeal whole-genome shotgun assemblies available at NCBI as of March 2015. For the bacterial and archaeal reads, the highest-identity match was chosen. If there were multiple top hits, the lowest common ancestor was determined. Taxonomic abundances in the form of read counts were normalized based on the average genome length per species bucket. Using KEGG release 73.1, reads whose genomic coordinates overlapped known KEGG orthologs (K numbers) were tabulated. Coding sequences from reference genomes that have not been specifically annotated by KEGG were aligned to all known KEGG orthologs. Any coding sequence that had >70% identity and >70% query coverage with respect to a known KEGG ortholog was assigned to that KEGG ortholog to create links between new genomes and entries in the KEGG database (termed “k-genes” in this paper). KEGG modules (M numbers) were calculated stepwise and identified as complete if 65% of the reaction steps were present per detected species and for the metagenome as a whole. Pathways were constructed for each taxon and metagenome by calculating the minimum set through MinPath resulting from presence of the gene orthologs. Pipeline code can be found at the following Web location: https://github.com/cmmr/puma-deprecated.

For the 14 of 135 sample duplicates, we selected one duplicate at random for the analytic sample. Alpha diversity measures (observed species/k-genes and Shannon index value for species/k-genes) were calculated using the “estimate_richness” function in the Phyloseq R package (35). Then, on the basis of the rarefaction curves for alpha diversity, we rarefied each sample to 2,000,000 reads, eliminating six samples from our analysis (a FIT tube sample frozen day-4, a no-solution sample frozen day-0, one 95% ethanol sample frozen day-0 and two frozen day-4, and a FOBT card sample frozen day-0). Beta diversity measures were calculated based on Bray-Curtis and Jaccard distance matrices generated separately from the composition of estimated species and k-genes using the vegan R package (36). Alpha and beta diversity estimates presented for subsequent analyses were based on rarefied data.

### Statistical analyses.

Statistical analyses were conducted using R, version 3.5.2, and SAS, version 9.4 (SAS Institute, Inc.). To identify possible outliers and check for sample mishandling/misclassification, we performed calculation using the Partitioning Around Medoids algorithm based on Jaccard and Bray-Curtis distance with the number of clusters set to 15 (the number of subjects), but all samples clustered by subject.

We tested for differences in alpha diversity (observed species/k-genes and the Shannon diversity index for species/k-genes) between collection methods by calculating least-squares means of the alpha diversity metrics using mixed-effects models, adjusted for time at ambient temperature, for each collection method. We considered samples without solution frozen immediately to represent the referent group.

We estimated the percentage of variability (*R*^2^) explained by subject, collection method, and day of freezing based on the Bray-Curtis and Jaccard distance matrices using the Adonis function in the vegan package in R to perform permutational multivariate analysis of variance (36).

We calculated intraclass correlation coefficients (ICCs) to evaluate stability and concordance using a mixed-effects model with a random intercept for subject and fixed effects for freezing on day-0 or day-4 (stability) or for collection method (concordance). We calculated ICCs based on the three most abundant phyla (square root transformed), observed species/k-genes, the Shannon index value for species/k-genes, and the top two principal-coordinate vectors of Bray-Curtis and Jaccard distance matrices for species composition. We also calculated the ICCs for the square root-transformed relative abundance of the top 20 most abundant species and k-genes and of the top 10 most abundant modules and pathways present in >50% of fecal samples. To calculate ICCs for stability at ambient temperature, for each fecal collection method, we compared one sample replicate frozen on day-0 to one frozen on day-4 for each participant. To calculate ICCs for concordance, we compared one replicate of samples without solution frozen day-0 (the putative “gold standard”) to one replicate from each of the other collection methods frozen day-0 for each participant. We calculated 95% confidence intervals using the bootstrap-technique to resample the study population with replacement over 1,000 iterations. We interpreted ICC values of <40% as representing a poor outcome and ICC values of ≥75% as representing an excellent outcome (37).

To assess the sensitivity of our stability and concordance estimates to various assumptions, we (i) calculated Spearman correlations (SCCs) to assess concordance and to compare the rank order of the metagenomic metrics to the “gold standard”; (ii) repeated our phylum, species, k-gene, module, and pathway analyses addressing the compositional nature of microbial data by using centered log ratio transformations, described previously (31); (iii) repeated the phylum, species, k-gene, module, and pathway analyses using untransformed relative abundances; and (iv) calculated concordance ICCs for the microbiome metrics for each pairwise comparison of the collection methods (e.g., for FOBT cards versus RNAlater).

### Data availability.

The data determined in this work are available in the National Center for Biotechnology (NCBI) Sequence Read Archive under accession number PRJNA606198.
