# Supplementary material for: Comparison of Methods To Collect Fecal Samples for Microbiome Studies Using Whole-Genome Shotgun Metagenomic Sequencing
Source: mSphere. 2020 Feb 26;5(1):e00827-19. doi: 10.1128/mSphere.00827-19 (PMC7045388; doi:10.1128/mSphere.00827-19)
Supplement: TABLE S1 [file mSphere.00827-19-st001.docx]

| **Characteristic** | **N (%)** | **Mean (untransformed) ± SD** |
| --- | --- | --- |
| Female | 8 (53.3) |  |
| Non-hispanic White | 13 (86.7) |  |
| Age |  | 37.5 ± 10.7 |
| Non-smoker | 15 (100) |  |
| Currently drinks alcohol | 13 (86.7) |  |
| Bowel movement at least once/day | 12 (80.0) |  |
| Vegan/vegetarian | 1 (0.07) |  |
