# Supplementary material for: Comparison of Methods To Collect Fecal Samples for Microbiome Studies Using Whole-Genome Shotgun Metagenomic Sequencing
Source: mSphere. 2020 Feb 26;5(1):e00827-19. doi: 10.1128/mSphere.00827-19 (PMC7045388; doi:10.1128/mSphere.00827-19)
Supplement: TABLE S2 [file mSphere.00827-19-st002.docx]

| **Sample collection type** | **Observed species** | |  | **Shannon index for species** | |  | **Observed k-genes** | |  | **Shannon Index for k-genes** | |  |
| --- | --- | --- | --- | --- | --- | --- | --- | --- | --- | --- | --- | --- |
|  | Adjusted Mean^a^ | 95% CI | *p ^b^* | Adjusted Mean^a^ | 95% CI | *p ^b^* | Adjusted Mean^a^ | 95% CI | *p ^b^* | Adjusted Mean^a^ | 95% CI | *p ^b^* |
| **95% ethanol** | 534 | (478, 590) | 0.001 | 2.86 | (2.71, 3.02) | 0.21 | 29,589 | (26,921, 32,258) | 0.34 | 8.60 | (8.50, 8.70) | 0.52 |
| **FIT tubes** | 549 | (493, 605) | 0.02 | 2.84 | (2.69, 3.00) | 0.12 | 29,592 | (26,930, 32,254) | 0.33 | 8.62 | (8.52, 8.72) | 0.20 |
| **FOBT cards** | 539 | (484, 595) | 0.002 | 2.97 | (2.82, 3.13) | 0.67 | 30,894 | (28,237, 33,552) | 0.40 | 8.68 | (8.58, 8.78) | 0.004 |
| **RNAlater** | 537 | (481, 592) | 0.001 | 2.75 | (2.60, 2.91) | 0.004 | 28,369 | (25,712, 31,027) | 0.009 | 8.51 | (8.41, 8.61) | 0.10 |
| **No Solution** | 580 | (522, 638) | ref | 2.95 | (2.77, 3.12) | ref | 30,290 | (27,483, 33,096) | ref | 8.57 | (8.46, 8.68) | ref |
| ^a^ Calculated from linear mixed effects models in Proc Glimmix (SAS) adjusting for day-0 or day-4 of freezing ^b^ Wald test p-value | | | | | | | | | | | |  |
