# Supplementary figures and images for: Comparison of Methods To Collect Fecal Samples for Microbiome Studies Using Whole-Genome Shotgun Metagenomic Sequencing
Source: mSphere. 2020 Feb 26;5(1):e00827-19. doi: 10.1128/mSphere.00827-19 (PMC7045388; doi:10.1128/mSphere.00827-19)

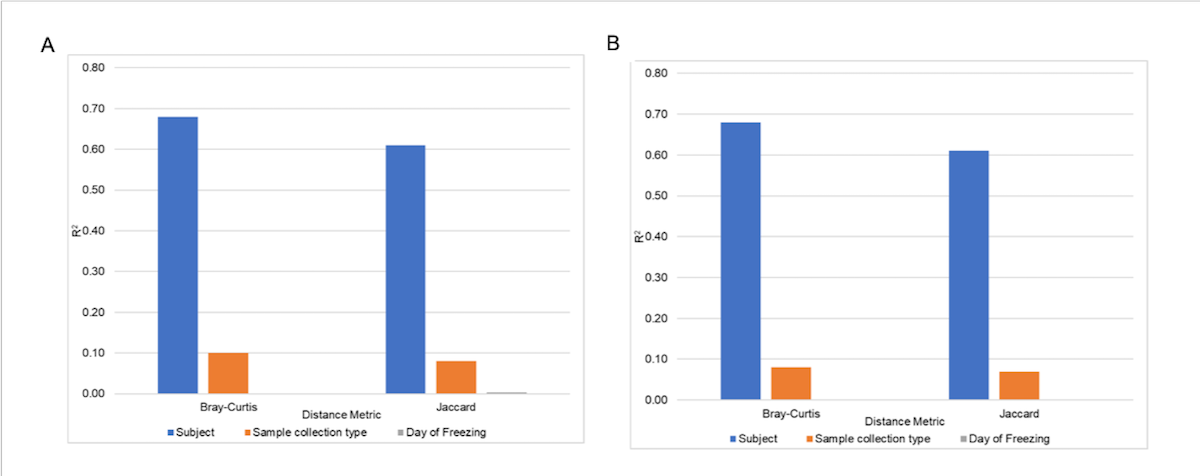

Supplement: FIG S1 [file mSphere.00827-19-sf001.tif]

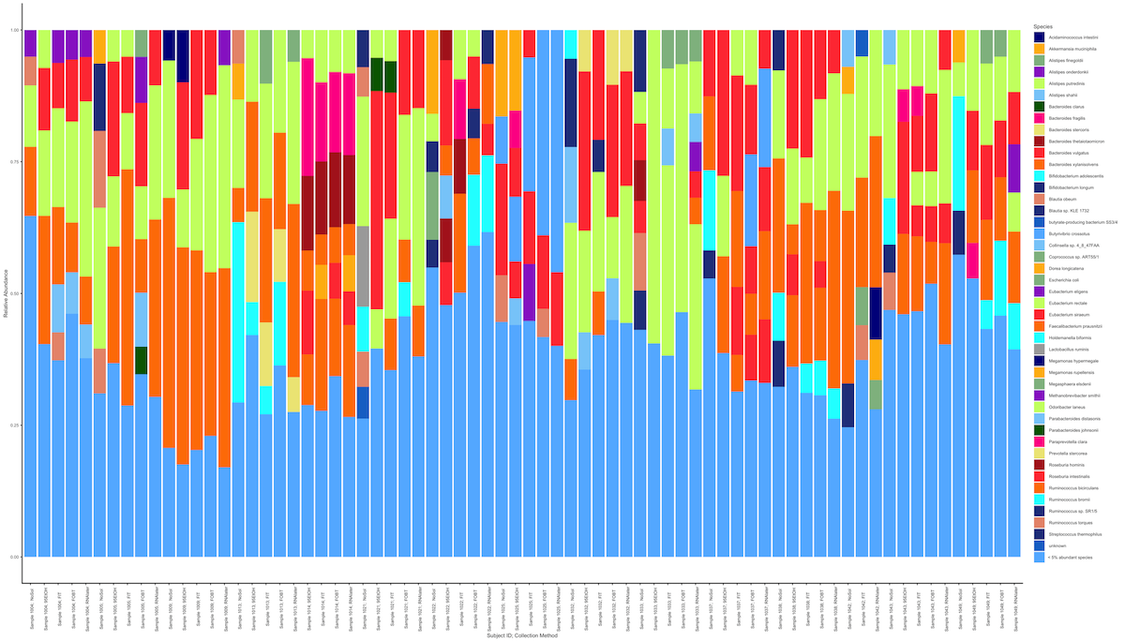

Supplement: FIG S2 [file mSphere.00827-19-sf002.tif]
