# Supplementary material for: Comparison of Methods To Collect Fecal Samples for Microbiome Studies Using Whole-Genome Shotgun Metagenomic Sequencing
Source: mSphere. 2020 Feb 26;5(1):e00827-19. doi: 10.1128/mSphere.00827-19 (PMC7045388; doi:10.1128/mSphere.00827-19)
Supplement: TABLE S3 [file mSphere.00827-19-st003.docx]

|  | **Species** | | | | |  | **K-genes** | | | | |
| --- | --- | --- | --- | --- | --- | --- | --- | --- | --- | --- | --- |
|  | **Bray-Curtis** | |  | **Jaccard** | |  | **Bray-Curtis** | |  | **Jaccard** | |
|  | **R^2*^** | **Pr(>F)** |  | **R^2*^** | **Pr(>F)** |  | **R^2*^** | **Pr(>F)** |  | **R^2*^** | **Pr(>F)** |
| **Subject** | 0.71 | 0.001 |  | 0.62 | 0.001 |  | 0.68 | 0.001 |  | 0.61 | 0.001 |
| **Sample collection type** | 0.09 | 0.002 |  | 0.07 | 0.001 |  | 0.10 | 0.001 |  | 0.08 | 0.001 |
| **Day of freezing** | 0.004 | 0.90 |  | 0.004 | 0.99 |  | 0.001 | 0.94 |  | 0.004 | 0.98 |
