# Supplementary material for: Comparison of Methods To Collect Fecal Samples for Microbiome Studies Using Whole-Genome Shotgun Metagenomic Sequencing
Source: mSphere. 2020 Feb 26;5(1):e00827-19. doi: 10.1128/mSphere.00827-19 (PMC7045388; doi:10.1128/mSphere.00827-19)
Supplement: TABLE S4 [file mSphere.00827-19-st004.docx]

|  | **95% Ethanol** | | |  | **FIT** | | |  | **FOBT** | | |  | **RNAlater** | | |
| --- | --- | --- | --- | --- | --- | --- | --- | --- | --- | --- | --- | --- | --- | --- | --- |
| **Microbiome metric** | **Day-0 Mean** | **Day-4 Mean** | **Stability ICC (95% CI)** |  | **Day-0 Mean** | **Day-4 Mean** | **Stability ICC (95% CI)** |  | **Day-0 Mean** | **Day-4 Mean** | **Stability ICC (95% CI)** |  | **Day-0 Mean** | **Day-4 Mean** | **Stability ICC (95% CI)** |
| Actinobacteria^a^ | 0.023 | 0.029 | 0.37 (0.00, 0.79) |  | 0.016 | 0.016 | 0.88 (0.70, 0.97) |  | 0.028 | 0.028 | 0.88 (0.68, 0.96) |  | 0.027 | 0.034 | 0.93 (0.77, 0.98) |
| Bacteroidetes^a^ | 0.486 | 0.321 | 0.42 (0.00, 0.80) |  | 0.387 | 0.329 | 0.93 (0.81, 0.98) |  | 0.399 | 0.385 | 0.94 (0.84, 0.98) |  | 0.274 | 0.250 | 0.96 (0.89, 0.99) |
| Firmicutes^a^ | 0.417 | 0.504 | 0.66 (0.19, 0.88) |  | 0.530 | 0.591 | 0.93 (0.81, 0.98) |  | 0.502 | 0.517 | 0.92 (0.78, 0.98) |  | 0.642 | 0.667 | 0.97 (0.92, 0.99) |
| Observed species | 525 | 532 | 0.93 (0.78, 0.98) |  | 540 | 537 | 0.90 (0.74, 0.97) |  | 543 | 537 | 0.99 (0.96, 1.00) |  | 523 | 546 | 0.98 (0.94, 0.99) |
| Observed genes | 29,564 | 29,263 | 0.77 (0.45, 0.93) |  | 29,624 | 28,603 | 0.86 (0.67, 0.96) |  | 31,495 | 30,843 | 0.93 (0.82, 0.98) |  | 28,289 | 28,788 | 0.95 (0.86, 0.99) |
| Shannon index for species | 2.86 | 2.81 | 0.57 (0.08, 0.85) |  | 2.84 | 2.78 | 0.88 (0.68, 0.96) |  | 3.00 | 2.97 | 0.97 (0.93, 0.99) |  | 2.77 | 2.78 | 0.93 (0.81, 0.98) |
| Shannon index for genes | 8.62 | 8.55 | 0.58 (0.10, 0.87) |  | 8.63 | 8.58 | 0.89 (0.67, 0.97) |  | 8.69 | 8.68 | 0.96 (0.89, 0.99) |  | 8.53 | 8.52 | 0.93 (0.80, 0.98) |
| BC Axis 1 | 0.02 | 0.02 | 0.73 (0.37, 0.92) |  | 0.01 | -0.01 | 0.99 (0.95, 1.00) |  | 0.05 | 0.04 | 0.98 (0.96, 1.00) |  | -0.02 | -0.02 | 0.99 (0.98, 1.00) |
| BC Axis 2 | -0.11 | -0.01 | 0.61 (0.18, 0.88) |  | -0.01 | 0.01 | 0.98 (0.93, 0.99) |  | -0.06 | -0.05 | 0.96 (0.88, 0.99) |  | 0.01 | 0.01 | 0.97 (0.93, 0.99) |
| JAC Axis 1 | -0.01 | -0.02 | 0.75 (0.43, 0.92) |  | -0.01 | 0.01 | 0.98 (0.95, 1.00) |  | -0.04 | -0.03 | 0.99 (0.96, 1.00) |  | 0.02 | 0.02 | 0.99 (0.98, 1.00) |
| JAC Axis 2 | -0.10 | -0.01 | 0.70 (0.25, 0.89) |  | 0.00 | 0.03 | 0.97 (0.91, 0.99) |  | -0.07 | -0.06 | 0.97 (0.92, 0.99) |  | 0.01 | 0.01 | 0.97 (0.92, 0.99) |
| ^a^ Phylum relative abundances were square root transformed prior to calculating ICCs | | | | | | | | | | | | | | | |
|  |  |  |  |  |  |  |  |  |  |  |  |  |  |  |  |
