# Supplementary material for: Comparison of Methods To Collect Fecal Samples for Microbiome Studies Using Whole-Genome Shotgun Metagenomic Sequencing
Source: mSphere. 2020 Feb 26;5(1):e00827-19. doi: 10.1128/mSphere.00827-19 (PMC7045388; doi:10.1128/mSphere.00827-19)
Supplement: TABLE S5 [file mSphere.00827-19-st005.docx]

|  | **95% Ethanol** | | | **FIT** | | | **FOBT** | | | **RNAlater** | | |
| --- | --- | --- | --- | --- | --- | --- | --- | --- | --- | --- | --- | --- |
| **Species** | **Day-0 Mean** | **Day-4 Mean** | **Stability ICC (95% CI)** | **Day-0 Mean** | **Day-4 Mean** | **Stability ICC (95% CI)** | **Day-0 Mean** | **Day-4 Mean** | **Stability ICC (95% CI)** | **Day-0 Mean** | **Day-4 Mean** | **Stability ICC (95% CI)** |
| Alistipes finegoldii | 0.016 | 0.022 | 0.67 (0.26, 0.89) | 0.018 | 0.019 | 0.99 (0.97, 1.00) | 0.026 | 0.023 | 0.88 (0.71, 0.96) | 0.017 | 0.016 | 0.99 (0.97, 1.00) |
| Alistipes putredinis | 0.041 | 0.062 | 0.56 (0.08, 0.85) | 0.049 | 0.037 | 0.96 (0.88, 0.99) | 0.047 | 0.052 | 0.86 (0.63, 0.96) | 0.038 | 0.038 | 0.99 (0.98, 1.00) |
| Alistipes shahii | 0.013 | 0.015 | 0.70 (0.30, 0.90) | 0.016 | 0.014 | 0.97 (0.92, 0.99) | 0.017 | 0.019 | 0.94 (0.85, 0.98) | 0.014 | 0.013 | 0.97 (0.92, 0.99) |
| Bacteroides fragilis | 0.029 | 0.021 | 0.85 (0.61, 0.96) | 0.029 | 0.022 | 0.85 (0.60, 0.95) | 0.023 | 0.023 | 0.91 (0.76, 0.97) | 0.016 | 0.014 | 0.99 (0.96, 1.00) |
| Bacteroides thetaiotaomicron | 0.020 | 0.017 | 0.81 (0.53, 0.94) | 0.026 | 0.024 | 0.93 (0.81, 0.98) | 0.020 | 0.021 | 0.95 (0.86, 0.98) | 0.015 | 0.013 | 0.97 (0.92, 0.99) |
| Bacteroides vulgatus | 0.163 | 0.082 | 0.68 (0.29, 0.90) | 0.106 | 0.086 | 0.96 (0.90, 0.99) | 0.106 | 0.104 | 0.97 (0.90, 0.99) | 0.073 | 0.068 | 0.98 (0.93, 0.99) |
| Bifidobacterium longum | 0.014 | 0.018 | 0.50 (0.00, 0.83) | 0.008 | 0.010 | 0.92 (0.79, 0.98) | 0.013 | 0.013 | 0.88 (0.68, 0.97) | 0.016 | 0.019 | 0.97 (0.91, 0.99) |
| Blautia obeum | 0.009 | 0.013 | 0.35 (0.00, 0.77) | 0.006 | 0.006 | 0.74 (0.39, 0.93) | 0.012 | 0.012 | 0.75 (0.43, 0.92) | 0.007 | 0.007 | 0.90 (0.73, 0.97) |
| Butyrivibrio crossotus | 0.010 | 0.017 | 0.17 (0.00, 0.66) | 0.024 | 0.027 | 1.00 (0.99, 1.00) | 0.041 | 0.039 | 1.00 (1.00, 1.00) | 0.047 | 0.045 | 1.00 (1.00 1.00) |
| Dorea longicatena | 0.007 | 0.013 | 0.68 (0.26, 0.91) | 0.014 | 0.012 | 0.90 (0.71, 0.97) | 0.007 | 0.007 | 0.90 (0.73, 0.97) | 0.012 | 0.012 | 0.95 (0.87, 0.99) |
| Eubacterium eligens | 0.018 | 0.012 | 0.58 (0.09, 0.86) | 0.006 | 0.006 | 0.93 (0.81, 0.98) | 0.023 | 0.020 | 0.82 (0.57, 0.94) | 0.028 | 0.030 | 0.60 (0.17, 0.86) |
| Eubacterium rectale | 0.093 | 0.141 | 0.69 (0.31, 0.89) | 0.110 | 0.115 | 0.95 (0.86, 0.99) | 0.114 | 0.111 | 0.96 (0.88, 0.99) | 0.177 | 0.184 | 0.99 (0.96, 1.00) |
| Eubacterium siraeum | 0.025 | 0.037 | 0.23 (0.00, 0.69) | 0.029 | 0.029 | 0.96 (0.89, 0.99) | 0.034 | 0.037 | 0.98 (0.93, 0.99) | 0.040 | 0.045 | 0.92 (0.76, 0.97) |
| Faecalibacterium prausnitzii | 0.101 | 0.112 | 0.80 (0.50, 0.93) | 0.158 | 0.184 | 0.92 (0.78, 0.98) | 0.094 | 0.100 | 0.95 (0.86, 0.99) | 0.136 | 0.119 | 0.89 (0.69, 0.97) |
| Odoribacter laneus | 0.046 | 0.029 | 0.97 (0.93, 0.99) | 0.028 | 0.026 | 1.00 (1.00, 1.00) | 0.027 | 0.028 | 1.00 (1.00, 1.00) | 0.022 | 0.020 | 1.00 (0.99, 1.00) |
| Parabacteroides distasonis | 0.020 | 0.016 | 0.06 (0.00, 0.62) | 0.015 | 0.011 | 0.90 (0.75, 0.97) | 0.028 | 0.020 | 0.53 (0.12, 0.82) | 0.014 | 0.010 | 0.93 (0.80, 0.98) |
| Roseburia intestinalis | 0.018 | 0.015 | 0.76 (0.43, 0.92) | 0.023 | 0.044 | 0.78 (0.46, 0.93) | 0.017 | 0.021 | 0.90 (0.74, 0.97) | 0.021 | 0.021 | 0.74 (0.38, 0.92) |
| Ruminococcus bicirculans | 0.050 | 0.034 | 0.78 (0.39, 0.93) | 0.045 | 0.047 | 0.99 (0.98, 1.00) | 0.030 | 0.040 | 0.90 (0.74, 0.97) | 0.050 | 0.061 | 0.97 (0.91, 0.99) |
| Ruminococcus bromii | 0.015 | 0.023 | 0.67 (0.29, 0.89) | 0.016 | 0.019 | 0.78 (0.49, 0.93) | 0.044 | 0.046 | 0.99 (0.97, 1.00) | 0.025 | 0.026 | 0.98 (0.95, 0.99) |
| Ruminococcus torques | 0.012 | 0.021 | 0.58 (0.10, 0.85) | 0.018 | 0.021 | 0.89 (0.66, 0.97) | 0.018 | 0.017 | 0.93 (0.82, 0.98) | 0.017 | 0.017 | 0.91 (0.76, 0.97) |
|  |  |  |  |  |  |  |  |  |  |  |  |  |
| **K-genes** |  |  |  |  |  |  |  |  |  |  |  |  |
| K00688; starch phosphorylase | 0.003 | 0.003 | 0.83 (0.55, 0.94) | 0.003 | 0.003 | 0.96 (0.89, 0.99) | 0.003 | 0.003 | 0.93 (0.81, 0.98) | 0.003 | 0.003 | 0.96 (0.91, 0.99) |
| K01190; beta-galactosidase | 0.007 | 0.006 | 0.39 (0.00, 0.79) | 0.007 | 0.007 | 0.74 (0.41, 0.92) | 0.007 | 0.007 | 0.87 (0.67, 0.96) | 0.006 | 0.006 | 0.94 (0.83, 0.98) |
| K01915; glutamine synthetase | 0.003 | 0.003 | 0.69 (0.32, 0.90) | 0.003 | 0.003 | 0.94 (0.81, 0.98) | 0.003 | 0.003 | 0.93 (0.78, 0.98) | 0.003 | 0.003 | 0.93 (0.80, 0.98) |
| K01955; carbamoyl-phosphate synthase large subunit | 0.003 | 0.003 | 0.68 (0.27, 0.90) | 0.003 | 0.003 | 0.96 (0.88, 0.99) | 0.003 | 0.003 | 0.92 (0.78, 0.98) | 0.003 | 0.003 | 0.95 (0.87, 0.99) |
| K01977; 16S ribosomal RNA | 0.004 | 0.004 | 0.84 (0.59, 0.95) | 0.004 | 0.004 | 0.84 (0.57, 0.95) | 0.004 | 0.004 | 0.96 (0.86, 0.99) | 0.004 | 0.004 | 0.97 (0.92, 0.99) |
| K01980; 23S ribosomal RNA | 0.009 | 0.009 | 0.84 (0.57, 0.95) | 0.009 | 0.009 | 0.90 (0.74, 0.97) | 0.009 | 0.009 | 0.98 (0.94, 0.99) | 0.009 | 0.010 | 0.97 (0.92, 0.99) |
| K01990; ABC-2 type transport system ATP-binding protein | 0.003 | 0.004 | 0.53 (0.00, 0.84) | 0.003 | 0.004 | 0.82 (0.52, 0.95) | 0.004 | 0.004 | 0.93 (0.81, 0.98) | 0.004 | 0.004 | 0.80 (0.49, 0.94) |
| K01992; ABC-2 type transport system permease protein | 0.004 | 0.004 | 0.60 (0.17, 0.86) | 0.004 | 0.003 | 0.57 (0.08, 0.87) | 0.004 | 0.004 | 0.92 (0.80, 0.98) | 0.004 | 0.004 | 0.91 (0.78, 0.97) |
| K02003; putative ABC transport system ATP-binding protein | 0.003 | 0.004 | 0.43 (0.00, 0.80) | 0.003 | 0.003 | 0.95 (0.86, 0.99) | 0.004 | 0.004 | 0.87 (0.68, 0.96) | 0.004 | 0.004 | 0.98 (0.93, 0.99) |
| K02004; putative ABC transport system permease protein | 0.008 | 0.009 | 0.48 (0.01, 0.81) | 0.008 | 0.008 | 0.93 (0.81, 0.98) | 0.009 | 0.009 | 0.90 (0.72, 0.97) | 0.009 | 0.009 | 0.97 (0.93, 0.99) |
| K02355; elongation factor G | 0.003 | 0.003 | 0.70 (0.31, 0.90) | 0.003 | 0.003 | 0.81 (0.53, 0.94) | 0.003 | 0.003 | 0.96 (0.88, 0.99) | 0.003 | 0.003 | 0.96 (0.89, 0.99) |
| K02469; DNA gyrase subunit A | 0.003 | 0.003 | 0.44 (0.00, 0.80) | 0.003 | 0.003 | 0.76 (0.42, 0.93) | 0.003 | 0.003 | 0.89 (0.70, 0.96) | 0.003 | 0.003 | 0.97 (0.92, 0.99) |
| K03043; DNA-directed RNA polymerase subunit beta | 0.004 | 0.004 | 0.83 (0.58, 0.95) | 0.004 | 0.004 | 0.92 (0.78, 0.98) | 0.004 | 0.004 | 0.95 (0.87, 0.99) | 0.004 | 0.004 | 0.90 (0.72, 0.97) |
| K03046; DNA-directed RNA polymerase subunit beta' | 0.004 | 0.004 | 0.91 (0.75, 0.98) | 0.004 | 0.004 | 0.90 (0.75, 0.97) | 0.004 | 0.004 | 0.92 (0.77, 0.98) | 0.003 | 0.004 | 0.96 (0.88, 0.99) |
| K03088; RNA polymerase sigma-70 factor, ECF subfamily | 0.006 | 0.005 | 0.38 (0.00, 0.77) | 0.005 | 0.005 | 0.82 (0.57, 0.94) | 0.005 | 0.005 | 0.89 (0.73, 0.97) | 0.004 | 0.004 | 0.96 (0.89, 0.99) |
| K03406; methyl-accepting chemotaxis protein | 0.002 | 0.003 | 0.74 (0.39, 0.92) | 0.003 | 0.004 | 0.84 (0.59, 0.95) | 0.003 | 0.003 | 0.88 (0.70, 0.96) | 0.004 | 0.004 | 0.95 (0.86, 0.98) |
| K03657; DNA helicase II / ATP-dependent DNA helicase PcrA | 0.003 | 0.004 | 0.73 (0.38, 0.91) | 0.003 | 0.003 | 0.72 (0.31, 0.92) | 0.003 | 0.004 | 0.89 (0.69, 0.97) | 0.004 | 0.004 | 0.92 (0.78, 0.97) |
| K05349; beta-glucosidase | 0.005 | 0.004 | 0.51 (0.03, 0.84) | 0.005 | 0.005 | 0.97 (0.91, 0.99) | 0.005 | 0.005 | 0.94 (0.83, 0.98) | 0.004 | 0.004 | 0.85 (0.60, 0.95) |
| K06147; ATP-binding cassette, subfamily B, bacterial | 0.008 | 0.009 | 0.08 (0.00, 0.61) | 0.009 | 0.010 | 0.79 (0.48, 0.94) | 0.009 | 0.009 | 0.79 (0.48, 0.92) | 0.010 | 0.011 | 0.90 (0.73, 0.97) |
| K07133; uncharacterized protein | 0.003 | 0.003 | 0.57 (0.10, 0.85) | 0.003 | 0.003 | 0.86 (0.64, 0.96) | 0.003 | 0.003 | 0.92 (0.78, 0.98) | 0.003 | 0.003 | 0.96 (0.88, 0.99) |
|  |  |  |  |  |  |  |  |  |  |  |  |  |
| **Modules** |  |  |  |  |  |  |  |  |  |  |  |  |
| M00001; glycolysis (Embden-Meyerhof pathway), glucose => pyruvate | 0.044 | 0.045 | 0.51 (0.04, 0.82) | 0.045 | 0.046 | 0.85 (0.57, 0.96) | 0.045 | 0.045 | 0.89 (0.69, 0.96) | 0.046 | 0.046 | 0.94 (0.86, 0.98) |
| M00002; glycolysis, core module involving three-carbon compounds | 0.025 | 0.027 | 0.47 (0.00, 0.83) | 0.026 | 0.026 | 0.78 (0.48, 0.93) | 0.026 | 0.026 | 0.97 (0.92, 0.99) | 0.027 | 0.027 | 0.97 (0.93, 0.99) |
| M00003; gluconeogenesis, oxaloacetate => fructose-6P | 0.035 | 0.037 | 0.78 (0.47, 0.94) | 0.036 | 0.037 | 0.89 (0.68, 0.97) | 0.036 | 0.036 | 0.95 (0.87, 0.99) | 0.038 | 0.038 | 0.98 (0.95, 0.99) |
| M00016; lysine biosynthesis, succinyl-DAP pathway, aspartate => lysine | 0.022 | 0.024 | 0.21 (0.00, 0.67) | 0.023 | 0.024 | 0.92 (0.80, 0.98) | 0.024 | 0.024 | 0.91 (0.78, 0.97) | 0.026 | 0.026 | 0.93 (0.82, 0.98) |
| M00048; inosine monophosphate biosynthesis, PRPP + glutamine => IMP | 0.036 | 0.036 | 0.51 (0.07, 0.83) | 0.035 | 0.036 | 0.94 (0.85, 0.98) | 0.036 | 0.037 | 0.97 (0.92, 0.99) | 0.036 | 0.036 | 0.98 (0.93, 0.99) |
| M00051; uridine monophosphate biosynthesis, glutamine (+ PRPP) => UMP | 0.031 | 0.032 | 0.36 (0.00, 0.75) | 0.031 | 0.032 | 0.94 (0.84, 0.98) | 0.032 | 0.032 | 0.87 (0.70, 0.96) | 0.033 | 0.033 | 0.83 (0.58, 0.94) |
| M00165; reductive pentose phosphate cycle (Calvin cycle) | 0.029 | 0.030 | 0.62 (0.15, 0.88) | 0.031 | 0.032 | 0.92 (0.80, 0.98) | 0.029 | 0.029 | 0.93 (0.82, 0.98) | 0.032 | 0.032 | 0.97 (0.91, 0.99) |
| M00167; reductive pentose phosphate cycle, glyceraldehyde-3P => ribulose-5P | 0.026 | 0.026 | 0.64 (0.23, 0.89) | 0.027 | 0.028 | 0.88 (0.66, 0.97) | 0.026 | 0.026 | 0.90 (0.75, 0.97) | 0.028 | 0.028 | 0.98 (0.95, 0.99) |
| M00173; reductive citrate cycle (Arnon-Buchanan cycle) | 0.037 | 0.033 | 0.43 (0.00, 0.79) | 0.035 | 0.032 | 0.83 (0.54, 0.95) | 0.033 | 0.032 | 0.91 (0.76, 0.97) | 0.029 | 0.028 | 0.95 (0.85, 0.98) |
| M00565; trehalose biosynthesis, D-glucose 1P => trehalose | 0.024 | 0.028 | 0.78 (0.40, 0.94) | 0.027 | 0.030 | 0.93 (0.80, 0.98) | 0.027 | 0.028 | 0.92 (0.75, 0.98) | 0.032 | 0.032 | 0.98 (0.93, 0.99) |
|  |  |  |  |  |  |  |  |  |  |  |  |  |
| **Pathways** |  |  |  |  |  |  |  |  |  |  |  |  |
| ko00230; purine metabolism | 0.036 | 0.036 | 0.90 (0.72, 0.97) | 0.035 | 0.035 | 0.89 (0.71, 0.97) | 0.036 | 0.036 | 0.91 (0.77, 0.97) | 0.035 | 0.036 | 0.84 (0.54, 0.95) |
| ko00520; amino sugar and nucleotide sugar metabolism | 0.020 | 0.020 | 0.52 (0.00, 0.83) | 0.020 | 0.020 | 0.90 (0.74, 0.97) | 0.020 | 0.020 | 0.87 (0.64, 0.96) | 0.019 | 0.019 | 0.82 (0.53, 0.94) |
| ko00970; aminoacyl-tRNA biosynthesis | 0.027 | 0.028 | 0.60 (0.10, 0.86) | 0.027 | 0.027 | 0.92 (0.76, 0.98) | 0.028 | 0.028 | 0.88 (0.69, 0.96) | 0.029 | 0.029 | 0.89 (0.70, 0.96) |
| ko01100; metabolic pathways | 0.250 | 0.246 | 0.75 (0.45, 0.92) | 0.245 | 0.247 | 0.73 (0.36, 0.91) | 0.249 | 0.250 | 0.56 (0.10, 0.84) | 0.248 | 0.247 | 0.71 (0.33, 0.91) |
| ko01110; biosynthesis of secondary metabolites | 0.077 | 0.075 | 0.36 (0.00, 0.76) | 0.077 | 0.076 | 0.65 (0.22, 0.88) | 0.080 | 0.079 | 0.39 (0.00, 0.78) | 0.078 | 0.075 | 0.65 (0.22, 0.88) |
| ko01120; microbial metabolism in diverse environments | 0.046 | 0.049 | 0.26 (0.00, 0.72) | 0.047 | 0.048 | 0.87 (0.66, 0.97) | 0.048 | 0.047 | 0.83 (0.62, 0.94) | 0.050 | 0.051 | 0.50 (0.00, 0.83) |
| ko01130; biosynthesis of antibiotics | 0.037 | 0.034 | 0.39 (0.00, 0.78) | 0.038 | 0.033 | 0.70 (0.30, 0.91) | 0.034 | 0.031 | 0.23 (0.00, 0.67) | 0.030 | 0.032 | 0.23 (0.00, 0.69) |
| ko01230; biosynthesis of amino acids | 0.030 | 0.030 | 0.43 (0.00, 0.82) | 0.029 | 0.030 | 0.79 (0.49, 0.94) | 0.030 | 0.030 | 0.65 (0.24, 0.88) | 0.031 | 0.031 | 0.65 (0.25, 0.87) |
| ko02010; ABC transporters | 0.025 | 0.029 | 0.66 (0.22, 0.88) | 0.027 | 0.030 | 0.79 (0.47, 0.93) | 0.027 | 0.028 | 0.90 (0.71, 0.97) | 0.031 | 0.031 | 0.93 (0.80, 0.98) |
| ko03010; ribosome | 0.028 | 0.031 | 0.86 (0.63, 0.96) | 0.028 | 0.028 | 0.92 (0.80, 0.98) | 0.028 | 0.028 | 0.96 (0.90, 0.99) | 0.029 | 0.030 | 0.95 (0.88, 0.99) |
