# Supplementary material for: Comparison of Methods To Collect Fecal Samples for Microbiome Studies Using Whole-Genome Shotgun Metagenomic Sequencing
Source: mSphere. 2020 Feb 26;5(1):e00827-19. doi: 10.1128/mSphere.00827-19 (PMC7045388; doi:10.1128/mSphere.00827-19)
Supplement: TABLE S6 [file mSphere.00827-19-st006.docx]

|  | **95% Ethanol** | | | | **FIT** | | | | **FOBT** | | | | **RNAlater** | | | |
| --- | --- | --- | --- | --- | --- | --- | --- | --- | --- | --- | --- | --- | --- | --- | --- | --- |
| **Microbiome Metric** | **95% Ethanol Mean** | **No-Solution Mean** | **Concordance ICC (95% CI)** | **Rs (95% CI)** | **FIT Mean** | **No-Solution Mean** | **Concordance ICC (95% CI)** | **Rs (95% CI)** | **FOBT Mean** | **No-Solution Mean** | **Concordance ICC (95% CI)** | **Rs (95% CI)** | **RNAlater Mean** | **No-Solution Mean** | **Concordance ICC (95% CI)** | **Rs (95% CI)** |
| Actinobacteria^a^ | 0.026 | 0.099 | 0.46 (0.00, 0.82) | 0.34 (0.18, 0.84) | 0.014 | 0.099 | 0.67 (0.25, 0.88) | 0.55 (0.18, 0.85) | 0.029 | 0.099 | 0.30 (0.00, 0.74) | 0.31 (0.18, 0.81) | 0.029 | 0.099 | 0.56 (0.08, 0.84) | 0.68 (0.18, 0.92) |
| Bacteroidetes^a^ | 0.483 | 0.059 | 0.23 (0.00, 0.71) | 0.29 (0.04, 0.77) | 0.393 | 0.059 | 0.56 (0.08, 0.83) | 0.64 (0.04, 0.91) | 0.389 | 0.063 | 0.36 (0.00, 0.76) | 0.45 (0.04, 0.82) | 0.245 | 0.059 | 0.52 (0.03, 0.82) | 0.58 (0.04, 0.84) |
| Firmicutes^a^ | 0.420 | 0.735 | 0.33 (0.00, 0.76) | 0.49 (0.23, 0.84) | 0.530 | 0.735 | 0.51 (0.01, 0.81) | 0.68 (0.23, 0.88) | 0.508 | 0.724 | 0.40 (0.00, 0.78) | 0.55 (0.23, 0.87) | 0.670 | 0.735 | 0.57 (0.10, 0.84) | 0.78 (0.23, 0.96) |
| Observed species | 536 | 587 | 0.81 (0.54, 0.94) | 0.80 (0.62, 0.95) | 561 | 587 | 0.84 (0.56, 0.95) | 0.80 (0.62, 0.96) | 552 | 587 | 0.92 (0.77, 0.98) | 0.87 (0.62, 0.99) | 535 | 587 | 0.91 (0.72, 0.97) | 0.91 (0.62, 1.00) |
| Observed genes | 30,939 | 31,281 | 0.41 (0.00, 0.80) | 0.45 (-0.23, 0.87) | 30,893 | 31,281 | 0.48 (0.00, 0.80) | 0.58 (-0.03, 0.87) | 32,443 | 31,580 | 0.66 (0.18, 0.89) | 0.79 (0.40, 0.95) | 28,801 | 31,281 | 0.68 (0.26, 0.89) | 0.73 (0.38, 0.91) |
| Shannon index for genes | 8.672 | 8.607 | 0.61 (0.19, 0.87) | 0.71 (0.18, 0.91) | 8.661 | 8.607 | 0.44 (0.00, 0.78) | 0.58 (0.13, 0.74) | 8.720 | 8.622 | 0.65 (0.16, 0.89) | 0.73 (0.34, 0.91) | 8.523 | 8.607 | 0.71 (0.30, 0.90) | 0.70 (0.27, 0.93) |
| Shannon index for species | 2.90 | 2.96 | 0.40 (0.00, 0.79) | 0.40 (0.25, 0.83) | 2.88 | 2.96 | 0.63 (0.18, 0.87) | 0.67 (0.25, 0.90) | 2.99 | 3.00 | 0.64 (0.15, 0.88) | 0.46 (0.25, 0.86) | 2.73 | 2.96 | 0.78 (0.43, 0.93) | 0.71 (0.25, 0.93) |
| BC Axis 1 | 0.01 | -0.06 | 0.67 (0.28, 0.89) | 0.65 (0.60, 0.96) | -0.01 | -0.06 | 0.89 (0.67, 0.96) | 0.89 (0.60, 0.97) | 0.03 | -0.05 | 0.84 (0.56, 0.95) | 0.86 (0.60, 0.98) | -0.06 | -0.06 | 0.94 (0.83, 0.98) | 0.92 (0.60, 1.00) |
| BC Axis 2 | -0.14 | 0.19 | 0.42 (0.00, 0.80) | 0.38 (-0.20, 0.78) | -0.05 | 0.19 | 0.33 (0.00, 0.73) | 0.35 (-0.20, 0.75) | -0.08 | 0.19 | 0.36 (0.00, 0.76) | 0.30 (-0.20, 0.75) | 0.01 | 0.19 | 0.50 (0.01, 0.81) | 0.58 (-0.20, 0.84) |
| JAC Axis 1 | 0.01 | 0.04 | 0.65 (0.24, 0.88) | 0.64 (0.59, 0.97) | 0.01 | 0.04 | 0.87 (0.64, 0.96) | 0.85 (0.59, 0.97) | -0.02 | 0.02 | 0.84 (0.57, 0.95) | 0.85 (0.59, 1.00) | 0.06 | 0.04 | 0.91 (0.73, 0.97) | 0.85 (0.59, 0.99) |
| JAC Axis 2 | -0.13 | 0.17 | 0.39 (0.00, 0.79) | 0.27 (0.05, 0.76) | -0.04 | 0.17 | 0.35 (0.00, 0.75) | 0.49 (0.05, 0.92) | -0.09 | 0.16 | 0.33 (0.00, 0.75) | 0.39 (0.05, 0.80) | 0.02 | 0.17 | 0.55 (0.07, 0.83) | 0.71 (0.05, 0.93) |
| ^a^ Phylum relative abundances were square root transformed prior to calculating ICCs | | | | | | | | | | | | | | | | |
|  |  |  |  |  |  |  |  |  |  |  |  |  |  |  |  |  |
|  |  |  |  |  |  |  |  |  |  |  |  |  |  |  |  |  |
