# Supplementary material for: Comparison of Methods To Collect Fecal Samples for Microbiome Studies Using Whole-Genome Shotgun Metagenomic Sequencing
Source: mSphere. 2020 Feb 26;5(1):e00827-19. doi: 10.1128/mSphere.00827-19 (PMC7045388; doi:10.1128/mSphere.00827-19)
Supplement: TABLE S7 [file mSphere.00827-19-st007.pdf]

	95% Ethanol				FIT				FOBT				RNAlater			
	95% Ethanol Mean (untransformed)	No-Solution Mean (untransformed)	ICC (95% CI)	Rs (95% CI)	FIT Mean (untransformed)	No-Solution Mean (untransformed)	ICC (95% CI)	Rs (95% CI)	FOBT Mean (untransformed)	No-Solution Mean (untransformed)	ICC (95% CI)	Rs (95% CI)	RNA Mean (untransformed)	No-Solution Mean (untransformed)	ICC (95% CI)	Rs (95% CI)
**Species**																
Alistipes finegoldii	0.017	0.007	0.32 (0.00, 0.75)	0.44 (0.32, 0.91)	0.024	0.007	0.51 (0.01, 0.81)	0.71 (0.32, 0.96)	0.028	0.008	0.51 (0.00, 0.83)	0.52 (0.32, 0.94)	0.017	0.007	0.64 (0.21, 0.87)	0.82 (0.32, 0.97)
Alistipes putredinis	0.040	0.010	0.42 (0.00, 0.80)	0.63 (0.47, 0.93)	0.050	0.010	0.63 (0.19, 0.87)	0.86 (0.47, 0.98)	0.045	0.011	0.59 (0.06, 0.87)	0.84 (0.47, 0.98)	0.032	0.010	0.65 (0.21, 0.87)	0.88 (0.47, 0.99)
Alistipes shahii	0.015	0.006	0.78 (0.48, 0.93)	0.92 (0.54, 0.99)	0.017	0.006	0.61 (0.15, 0.86)	0.87 (0.54, 0.99)	0.017	0.006	0.65 (0.17, 0.89)	0.74 (0.54, 0.95)	0.013	0.006	0.72 (0.32, 0.90)	0.91 (0.54, 1.00)
Bacteroides fragilis	0.013	0.003	0.13 (0.00, 0.66)	0.45 (0.13, 0.83)	0.019	0.003	0.35 (0.00, 0.75)	0.67 (0.13, 0.93)	0.013	0.003	0.34 (0.00, 0.75)	0.60 (0.13, 0.92)	0.005	0.003	0.67 (0.24, 0.88)	0.72 (0.13, 0.93)
Bacteroides thetaiotaomicron	0.013	0.002	0.38 (0.00, 0.78)	0.51 (0.23, 0.90)	0.015	0.002	0.53 (0.04, 0.82)	0.74 (0.23, 0.97)	0.010	0.003	0.53 (0.00, 0.84)	0.57 (0.23, 0.91)	0.006	0.002	0.70 (0.29, 0.90)	0.75 (0.23, 0.95)
Bacteroides vulgatus	0.175	0.013	0.26 (0.00, 0.73)	0.25 (-0.26, 0.73)	0.115	0.013	0.39 (0.00, 0.76)	0.48 (-0.26, 0.84)	0.114	0.014	0.45 (0.00, 0.80)	0.21 (-0.26, 0.73)	0.073	0.013	0.46 (0.00, 0.80)	0.53 (-0.26, 0.85)
Bifidobacterium longum	0.015	0.045	0.53 (0.08, 0.84)	0.60 (0.51, 0.98)	0.008	0.045	0.63 (0.18, 0.87)	0.95 (0.51, 1.00)	0.014	0.048	0.56 (0.02, 0.86)	0.52 (0.51, 0.98)	0.016	0.045	0.72 (0.33, 0.90)	0.90 (0.51, 1.00)
Blautia obeum	0.010	0.042	0.50 (0.04, 0.83)	0.75 (0.08, 0.90)	0.005	0.042	0.33 (0.00, 0.73)	0.73 (0.08, 0.94)	0.012	0.044	0.02 (0.00, 0.56)	0.31 (0.08, 0.87)	0.006	0.042	0.30 (0.00, 0.71)	0.69 (0.08, 0.95)
Butyrivibrio crossotus	0.010	0.010	0.85 (0.61, 0.95)	0.08 (-0.13, 0.66)	0.022	0.010	0.87 (0.62, 0.96)	0.42 (-0.13, 0.87)	0.044	0.010	0.72 (0.30, 0.91)	0.43 (-0.13, 0.96)	0.047	0.010	0.69 (0.28, 0.89)	0.64 (-0.13, 0.97)
Dorea longicatena	0.004	0.025	0.48 (0.02, 0.82)	0.77 (0.17, 0.96)	0.009	0.025	0.62 (0.17, 0.86)	0.70 (0.17, 0.94)	0.006	0.023	0.51 (0.00, 0.83)	0.43 (0.17, 0.87)	0.008	0.025	0.59 (0.13, 0.85)	0.71 (0.17, 0.93)
Eubacterium rectale	0.093	0.152	0.49 (0.03, 0.83)	0.39 (0.25, 0.87)	0.117	0.152	0.81 (0.48, 0.94)	0.71 (0.25, 0.92)	0.122	0.147	0.74 (0.33, 0.92)	0.69 (0.25, 0.92)	0.195	0.152	0.91 (0.72, 0.97)	0.77 (0.25, 0.97)
Eubacterium siraeum	0.017	0.034	0.52 (0.07, 0.84)	0.65 (0.60, 0.94)	0.027	0.034	0.92 (0.75, 0.97)	0.93 (0.60, 1.00)	0.032	0.037	0.90 (0.72, 0.97)	0.84 (0.60, 0.98)	0.037	0.034	0.92 (0.76, 0.98)	0.92 (0.60, 1.00)
Eubacterium eligens	0.018	0.007	0.57 (0.13, 0.86)	0.55 (0.54, 0.91)	0.010	0.007	0.71 (0.30, 0.90)	0.79 (0.54, 0.97)	0.024	0.008	0.66 (0.18, 0.89)	0.63 (0.54, 0.95)	0.030	0.007	0.68 (0.26, 0.89)	0.87 (0.54, 0.99)
Faecalibacterium prausnitzii	0.098	0.093	0.62 (0.20, 0.87)	0.38 (-0.52, 0.80)	0.153	0.093	0.29 (0.00, 0.71)	0.23 (-0.52, 0.83)	0.090	0.075	0.40 (0.00, 0.78)	0.19 (-0.52, 0.71)	0.147	0.093	0.39 (0.00, 0.76)	0.22 (-0.52, 0.71)
Odoribacter laneus	0.046	0.002	0.34 (0.00, 0.77)	0.49 (0.25, 0.83)	0.026	0.002	0.45 (0.00, 0.79)	0.65 (0.25, 0.87)	0.029	0.002	0.44 (0.00, 0.80)	0.62 (0.25, 0.86)	0.022	0.002	0.48 (0.00, 0.80)	0.75 (0.25, 0.92)
Parabacteroides distasonis	0.026	0.006	0.25 (0.00, 0.72)	0.31 (0.57, 0.90)	0.021	0.006	0.78 (0.42, 0.92)	0.81 (0.57, 0.97)	0.030	0.007	0.36 (0.00, 0.76)	0.27 (0.57, 0.83)	0.014	0.006	0.93 (0.80, 0.98)	0.93 (0.57, 1.00)
Roseburia intestinalis	0.024	0.018	0.27 (0.00, 0.73)	0.57 (-0.06, 0.88)	0.022	0.018	0.31 (0.00, 0.72)	0.46 (-0.06, 0.92)	0.018	0.019	0.33 (0.00, 0.75)	0.50 (-0.06, 0.95)	0.023	0.018	0.27 (0.00, 0.70)	0.65 (-0.06, 0.94)
Ruminococcus torques	0.012	0.045	0.44 (0.00, 0.81)	0.71 (-0.19, 0.93)	0.021	0.045	0.27 (0.00, 0.70)	0.38 (-0.19, 0.76)	0.019	0.046	0.46 (0.00, 0.81)	0.51 (-0.19, 0.84)	0.018	0.045	0.47 (0.00, 0.80)	0.52 (-0.19, 0.90)
Ruminococcus bicirculans	0.050	0.033	0.73 (0.38, 0.91)	0.87 (0.57, 0.98)	0.046	0.033	0.86 (0.61, 0.96)	0.85 (0.57, 0.98)	0.032	0.035	0.95 (0.86, 0.99)	0.90 (0.57, 1.00)	0.051	0.033	0.90 (0.71, 0.97)	0.88 (0.57, 0.98)
Ruminococcus bromii	0.018	0.069	0.71 (0.34, 0.91)	0.95 (0.50, 1.00)	0.020	0.069	0.58 (0.12, 0.85)	0.82 (0.50, 0.95)	0.048	0.074	0.87 (0.63, 0.96)	0.94 (0.50, 1.00)	0.027	0.069	0.61 (0.15, 0.86)	0.88 (0.50, 0.99)
**K-genes**																
K00688; starch phosphorylase	0.003	0.003	0.77 (0.46, 0.93)	0.84 (0.52, 0.93)	0.003	0.003	0.82 (0.50, 0.94)	0.80 (0.48, 0.93)	0.003	0.003	0.78 (0.44, 0.93)	0.79 (0.45, 0.94)	0.004	0.003	0.91 (0.73, 0.97)	0.89 (0.61, 0.98)
K01190; beta-galactosidase	0.007	0.004	0.28 (0.00, 0.74)	0.13 (-0.60, 0.75)	0.007	0.004	0.28 (0.00, 0.71)	0.37 (-0.17, 0.74)	0.006	0.004	0.36 (0.00, 0.76)	0.29 (-0.28, 0.81)	0.006	0.004	0.49 (0.00, 0.81)	0.36 (-0.22, 0.77)
K01915; glutamine synthetase	0.003	0.003	0.12 (0.00, 0.66)	0.31 (-0.34, 0.82)	0.003	0.003	0.36 (0.00, 0.75)	0.39 (-0.27, 0.92)	0.003	0.003	0.24 (0.00, 0.71)	0.30 (-0.42, 0.94)	0.003	0.003	0.39 (0.00, 0.76)	0.37 (-0.26, 0.87)
K01955; carbamoyl-phosphate synthase large subunit	0.003	0.003	0.31 (0.00, 0.75)	-0.01 (-0.65, 0.60)	0.003	0.003	0.55 (0.07, 0.83)	0.02 (-0.70, 0.66)	0.003	0.003	0.71 (0.28, 0.91)	0.33 (-0.33, 0.88)	0.003	0.003	0.70 (0.28, 0.89)	0.34 (-0.30, 0.86)
K01977; 16S ribosomal RNA	0.005	0.004	0.54 (0.10, 0.85)	0.41 (-0.30, 0.86)	0.005	0.004	0.81 (0.48, 0.94)	0.83 (0.40, 0.98)	0.004	0.004	0.65 (0.18, 0.89)	0.86 (0.50, 0.99)	0.005	0.004	0.70 (0.28, 0.89)	0.89 (0.58, 0.98)
K01980; 23S ribosomal RNA	0.009	0.008	0.61 (0.19, 0.87)	0.68 (0.17, 0.94)	0.010	0.008	0.84 (0.54, 0.95)	0.81 (0.44, 0.96)	0.009	0.008	0.81 (0.49, 0.94)	0.87 (0.47, 1.00)	0.010	0.008	0.76 (0.40, 0.92)	0.82 (0.46, 0.97)
K01990; ABC-2 type transport system ATP-binding protein	0.003	0.004	0.66 (0.26, 0.89)	0.76 (0.23, 0.97)	0.003	0.004	0.71 (0.30, 0.90)	0.69 (0.12, 0.93)	0.004	0.004	0.76 (0.39, 0.93)	0.67 (0.04, 0.98)	0.004	0.004	0.63 (0.18, 0.87)	0.65 (0.06, 0.92)
K01992; ABC-2 type transport system permease protein	0.004	0.004	0.16 (0.00, 0.68)	0.26 (-0.39, 0.76)	0.003	0.004	0.08 (0.00, 0.59)	0.21 (-0.40, 0.78)	0.004	0.004	0.07 (0.00, 0.61)	0.21 (-0.52, 0.75)	0.004	0.004	Did not converge	0.07 (-0.53, 0.68)
K02003; putative ABC transport system ATP-binding protein	0.003	0.004	0.62 (0.20, 0.87)	0.59 (0.09, 0.91)	0.003	0.004	0.67 (0.25, 0.88)	0.60 (0.04, 0.95)	0.004	0.004	0.46 (0.00, 0.81)	0.42 (-0.36, 0.91)	0.004	0.004	0.63 (0.19, 0.87)	0.60 (-0.05, 0.97)
K02004; putative ABC transport system permease protein	0.008	0.009	Did not converge	-0.07 (-0.66, 0.60)	0.008	0.009	0.34 (0.00, 0.74)	0.35 (-0.25, 0.89)	0.009	0.009	Did not converge	-0.15 (-0.74, 0.52)	0.009	0.009	0.41 (0.00, 0.77)	0.31 (-0.31, 0.83)
K02355; elongation factor G	0.003	0.002	0.31 (0.00, 0.75)	0.25 (-0.41, 0.73)	0.003	0.002	0.34 (0.00, 0.74)	0.42 (-0.21, 0.84)	0.003	0.002	0.40 (0.00, 0.78)	0.32 (-0.29, 0.78)	0.003	0.002	0.55 (0.06, 0.83)	0.49 (-0.16, 0.86)
K02469; DNA gyrase subunit A	0.003	0.003	0.43 (0.00, 0.80)	0.55 (0.03, 0.86)	0.003	0.003	0.53 (0.05, 0.83)	0.68 (0.28, 0.89)	0.003	0.003	0.13 (0.00, 0.65)	0.29 (-0.41, 0.83)	0.003	0.003	0.07 (0.00, 0.58)	0.30 (-0.38, 0.91)
K03043; DNA-directed RNA polymerase subunit beta	0.004	0.003	0.52 (0.08, 0.84)	0.52 (-0.12, 0.85)	0.004	0.003	0.33 (0.00, 0.73)	0.48 (-0.19, 0.92)	0.004	0.003	0.25 (0.00, 0.71)	0.50 (-0.18, 0.92)	0.004	0.003	0.28 (0.00, 0.71)	0.47 (-0.19, 0.84)
K03046; DNA-directed RNA polymerase subunit beta'	0.004	0.004	0.35 (0.00, 0.77)	0.43 (-0.09, 0.80)	0.004	0.004	0.33 (0.00, 0.73)	0.35 (-0.34, 0.85)	0.004	0.004	0.14 (0.00, 0.66)	0.14 (-0.49, 0.67)	0.003	0.004	0.06 (0.00, 0.58)	0.13 (-0.49, 0.67)
K03088; RNA polymerase sigma-70 factor, ECF subfamily	0.006	0.002	Did not converge	-0.02 (-0.60, 0.63)	0.005	0.002	0.27 (0.00, 0.70)	0.41 (-0.23, 0.81)	0.005	0.002	0.22 (0.00, 0.70)	0.19 (-0.53, 0.68)	0.004	0.002	0.37 (0.00, 0.75)	0.46 (-0.17, 0.85)
K03406; methyl-accepting chemotaxis protein	0.003	0.003	0.20 (0.00, 0.69)	0.18 (-0.43, 0.71)	0.003	0.003	0.29 (0.00, 0.71)	0.26 (-0.40, 0.77)	0.003	0.003	Did not converge	-0.13 (-0.63, 0.40)	0.004	0.003	0.41 (0.00, 0.77)	0.48 (-0.01, 0.71)
K03657; DNA helicase II / ATP-dependent DNA helicase PcrA	0.003	0.004	0.21 (0.00, 0.70)	0.14 (-0.39, 0.73)	0.003	0.004	Did not converge	-0.07 (-0.71, 0.56)	0.004	0.004	0.37 (0.00, 0.77)	0.43 (-0.29, 0.94)	0.004	0.004	0.31 (0.00, 0.72)	0.23 (-0.41, 0.77)
K05349; beta-glucosidase	0.004	0.003	0.00 (0.00, 0.58)	0.04 (-0.62, 0.70)	0.005	0.003	Did not converge	0.07 (-0.57, 0.74)	0.004	0.003	0.28 (0.00, 0.72)	0.27 (-0.38, 0.72)	0.004	0.003	0.15 (0.00, 0.63)	0.22 (-0.42, 0.71)
K06147; ATP-binding cassette, subfamily B, bacterial	0.008	0.011	0.08 (0.00, 0.62)	0.14 (-0.46, 0.72)	0.009	0.011	0.21 (0.00, 0.67)	0.35 (-0.18, 0.73)	0.008	0.011	0.06 (0.00, 0.60)	0.10 (-0.55, 0.78)	0.011	0.011	0.35 (0.00, 0.74)	0.40 (-0.17, 0.82)
K07133; uncharacterized protein	0.003	0.003	0.34 (0.00, 0.77)	0.20 (-0.50, 0.74)	0.003	0.003	0.36 (0.00, 0.75)	0.37 (-0.29, 0.83)	0.003	0.003	0.50 (0.00, 0.83)	0.42 (-0.32, 0.90)	0.003	0.003	0.57 (0.09, 0.84)	0.62 (-0.03, 0.93)
**Modules**																
M00001; glycolysis (Embden-Meyerhof pathway), glucose => pyruvate	0.044	0.047	Did not converge	-0.17 (-0.75, 0.40)	0.045	0.047	0.32 (0.00, 0.72)	0.24 (-0.43, 0.75)	0.045	0.047	0.16 (0.00, 0.67)	0.22 (-0.43, 0.73)	0.046	0.047	0.33 (0.00, 0.73)	0.35 (-0.33, 0.82)
M00002; glycolysis, core module involving three-carbon compounds	0.026	0.028	0.06 (0.00, 0.61)	0.08 (-0.51, 0.56)	0.026	0.028	0.60 (0.13, 0.85)	0.35 (-0.32, 0.80)	0.026	0.028	0.41 (0.00, 0.79)	0.53 (0.02, 0.85)	0.027	0.028	0.50 (0.00, 0.81)	0.57 (0.07, 0.87)
M00003; gluconeogenesis, oxaloacetate => fructose-6P	0.035	0.036	0.16 (0.00, 0.68)	0.15 (-0.53, 0.79)	0.037	0.036	0.50 (0.01, 0.81)	0.47 (-0.15, 0.83)	0.037	0.036	0.63 (0.14, 0.88)	0.74 (0.23, 0.93)	0.039	0.036	0.76 (0.40, 0.92)	0.86 (0.65, 0.95)
M00016; lysine biosynthesis, succinyl-DAP pathway, aspartate => lysine	0.023	0.028	0.22 (0.00, 0.71)	0.13 (-0.60, 0.76)	0.023	0.028	0.33 (0.00, 0.73)	0.36 (-0.35, 0.78)	0.024	0.028	Did not converge	-0.06 (-0.67, 0.49)	0.026	0.028	0.44 (0.00, 0.79)	0.36 (-0.23, 0.74)
M00048; inosine monophosphate biosynthesis, PRPP + glutamine => IMP	0.036	0.037	0.19 (0.00, 0.69)	0.14 (-0.50, 0.62)	0.035	0.037	0.31 (0.00, 0.72)	0.21 (-0.41, 0.75)	0.037	0.037	0.46 (0.00, 0.81)	0.49 (-0.16, 0.86)	0.036	0.037	0.31 (0.00, 0.72)	0.31 (-0.34, 0.76)
M00051; uridine monophosphate biosynthesis, glutamine (+ PRPP) => UMP	0.031	0.034	0.00 (0.00, 0.58)	-0.05 (-0.52, 0.51)	0.031	0.034	0.03 (0.00, 0.56)	0.04 (-0.50, 0.53)	0.032	0.034	Did not converge	-0.23 (-0.89, 0.40)	0.033	0.034	Did not converge	-0.31 (-0.74, 0.20)
M00165; reductive pentose phosphate cycle (Calvin cycle)	0.029	0.029	0.57 (0.13, 0.86)	0.59 (-0.07, 0.92)	0.031	0.029	0.74 (0.36, 0.91)	0.75 (0.34, 0.97)	0.029	0.029	0.71 (0.28, 0.91)	0.71 (0.24, 0.98)	0.033	0.029	0.72 (0.32, 0.90)	0.81 (0.50, 0.96)
M00167; reductive pentose phosphate cycle, glyceraldehyde-3P => ribulose-5P	0.026	0.026	0.14 (0.00, 0.67)	-0.18 (-0.73, 0.49)	0.027	0.026	0.55 (0.07, 0.83)	0.43 (-0.22, 0.90)	0.026	0.026	0.42 (0.00, 0.79)	0.33 (-0.39, 0.81)	0.029	0.026	0.63 (0.18, 0.87)	0.64 (0.11, 0.91)
M00173; reductive citrate cycle (Arnon-Buchanan cycle)	0.036	0.022	0.38 (0.00, 0.78)	0.14 (-0.60, 0.76)	0.034	0.022	0.49 (0.00, 0.80)	0.50 (-0.06, 0.84)	0.032	0.023	0.16 (0.00, 0.67)	0.08 (-0.62, 0.58)	0.027	0.022	0.40 (0.00, 0.77)	0.46 (-0.13, 0.86)
M00565; trehalose biosynthesis, D-glucose 1P => trehalose	0.025	0.031	0.63 (0.22, 0.88)	0.64 (0.04, 0.95)	0.028	0.031	0.80 (0.47, 0.94)	0.79 (0.40, 0.98)	0.027	0.030	0.74 (0.34, 0.92)	0.81 (0.44, 0.98)	0.033	0.031	0.94 (0.81, 0.98)	0.88 (0.55, 0.98)
**Pathways**																
ko00230; purine metabolism	0.036	0.037	0.51 (0.05, 0.83)	0.66 (0.13, 0.93)	0.035	0.037	0.49 (0.00, 0.81)	0.49 (-0.21, 0.92)	0.036	0.037	0.25 (0.00, 0.71)	0.29 (-0.40, 0.91)	0.035	0.037	0.13 (0.00, 0.62)	0.20 (-0.46, 0.74)
ko00520; amino sugar and nucleotide sugar metabolism	0.020	0.019	0.09 (0.00, 0.64)	-0.17 (-0.69, 0.63)	0.020	0.019	0.41 (0.00, 0.77)	0.35 (-0.30, 0.87)	0.020	0.019	0.15 (0.00, 0.67)	0.09 (-0.56, 0.63)	0.019	0.019	0.29 (0.00, 0.71)	0.31 (-0.27, 0.73)
ko00970; aminoacyl-tRNA biosynthesis	0.027	0.029	0.33 (0.00, 0.76)	0.47 (-0.09, 0.84)	0.027	0.029	0.56 (0.07, 0.83)	0.56 (-0.02, 0.86)	0.028	0.029	0.09 (0.00, 0.62)	0.27 (-0.41, 0.72)	0.029	0.029	0.08 (0.00, 0.58)	0.23 (-0.40, 0.73)
ko01100; metabolic pathways	0.249	0.240	0.22 (0.00, 0.71)	0.32 (-0.32, 0.81)	0.245	0.240	0.20 (0.00, 0.67)	0.31 (-0.27, 0.72)	0.248	0.241	0.02 (0.00, 0.57)	-0.14 (-0.67, 0.39)	0.246	0.240	0.00 (0.00, 0.53)	0.04 (-0.45, 0.55)
ko01110; biosynthesis of secondary metabolites	0.077	0.078	Did not converge	-0.34 (-0.70, 0.17)	0.076	0.078	Did not converge	-0.10 (-0.64, 0.64)	0.079	0.079	Did not converge	-0.34 (-0.86, 0.37)	0.078	0.078	0.09 (0.00, 0.59)	0.29 (-0.40, 0.83)
ko01120; microbial metabolism in diverse environments	0.045	0.053	Did not converge	-0.08 (-0.68, 0.65)	0.046	0.053	0.02 (0.00, 0.55)	0.05 (-0.61, 0.68)	0.047	0.054	0.41 (0.00, 0.78)	0.48 (-0.19, 0.90)	0.051	0.053	Did not converge	-0.24 (-0.75, 0.47)
ko01130; biosynthesis of antibiotics	0.040	0.029	0.55 (0.10, 0.85)	0.62 (0.06, 0.91)	0.041	0.029	0.08 (0.00, 0.58)	-0.20 (-0.74, 0.49)	0.036	0.027	Did not converge	-0.01 (-0.63, 0.53)	0.030	0.029	0.10 (0.00, 0.60)	0.03 (-0.49, 0.63)
ko01230; biosynthesis of amino acids	0.030	0.032	0.20 (0.00, 0.70)	0.26 (-0.44, 0.81)	0.029	0.032	0.35 (0.00, 0.74)	0.46 (-0.14, 0.83)	0.030	0.032	Did not converge	-0.08 (-0.63, 0.58)	0.031	0.032	Did not converge	-0.13 (-0.75, 0.45)
ko02010; ABC transporters	0.026	0.035	0.29 (0.00, 0.74)	0.13 (-0.55, 0.73)	0.028	0.035	0.34 (0.00, 0.74)	0.40 (-0.13, 0.76)	0.028	0.035	0.46 (0.00, 0.81)	0.43 (-0.19, 0.83)	0.032	0.035	0.56 (0.08, 0.83)	0.68 (0.23, 0.89)
ko03010; ribosome	0.028	0.028	0.32 (0.00, 0.76)	0.32 (-0.30, 0.80)	0.029	0.028	0.72 (0.33, 0.90)	0.75 (0.37, 0.96)	0.029	0.028	0.57 (0.03, 0.86)	0.49 (-0.12, 0.89)	0.030	0.028	0.62 (0.17, 0.86)	0.60 (0.04, 0.92)
