# Supplementary material for: Comparison of Methods To Collect Fecal Samples for Microbiome Studies Using Whole-Genome Shotgun Metagenomic Sequencing
Source: mSphere. 2020 Feb 26;5(1):e00827-19. doi: 10.1128/mSphere.00827-19 (PMC7045388; doi:10.1128/mSphere.00827-19)
Supplement: TABLE S8 [file mSphere.00827-19-st008.docx]

|  | **95% Ethanol vs. FIT** | | | **95% Ethanol vs. FOBT** | | | **95% Ethanol vs. RNAlater** | | |
| --- | --- | --- | --- | --- | --- | --- | --- | --- | --- |
| **Microbiome metric** | **95% Ethanol Mean** | **FIT Mean** | **ICC (95% CI)** | **95% Ethanol Mean** | **FOBT Mean** | **ICC (95% CI)** | **95% Ethanol Mean** | **RNAlater Mean** | **ICC (95% CI)** |
| Actinobacteria^a^ | 0.026 | 0.014 | 0.65 (0.26, 0.89) | 0.026 | 0.029 | 0.32 (0.00, 0.74) | 0.026 | 0.029 | 0.53 (0.00, 0.84) |
| Bacteroidetes^a^ | 0.483 | 0.393 | 0.87 (0.68, 0.96) | 0.483 | 0.389 | 0.71 (0.27, 0.91) | 0.483 | 0.245 | 0.81 (0.49, 0.94) |
| Firmicutes^a^ | 0.420 | 0.530 | 0.85 (0.64, 0.95) | 0.420 | 0.508 | 0.66 (0.19, 0.89) | 0.420 | 0.670 | 0.79 (0.44, 0.94) |
| Observed species | 536 | 561 | 0.90 (0.75, 0.97) | 536 | 552 | 0.87 (0.65, 0.96) | 536 | 535 | 0.81 (0.50, 0.94) |
| Observed genes | 30,939 | 30,893 | 0.75 (0.42, 0.92) | 30,939 | 32,443 | 0.74 (0.34, 0.92) | 30,939 | 28,801 | 0.73 (0.31, 0.92) |
| Shannon index for species | 8.672 | 8.661 | 0.70 (0.33, 0.90) | 8.672 | 8.720 | 0.69 (0.24, 0.90) | 8.672 | 8.523 | 0.47 (0.00, 0.81) |
| Shannon index for genes | 2.90 | 2.88 | 0.66 (0.27, 0.89) | 2.90 | 2.99 | 0.73 (0.31, 0.92) | 2.90 | 2.73 | 0.61 (0.10, 0.87) |
| BC Axis 1 | 0.01 | -0.01 | 0.87 (0.68, 0.96) | 0.01 | 0.03 | 0.73 (0.31, 0.92) | 0.01 | -0.06 | 0.72 (0.30, 0.91) |
| BC Axis 2 | -0.14 | -0.05 | 0.87 (0.68, 0.96) | -0.14 | -0.08 | 0.86 (0.60, 0.96) | -0.14 | 0.01 | 0.78 (0.44, 0.93) |
| JAC Axis 1 | 0.01 | 0.01 | 0.84 (0.61, 0.95) | 0.01 | -0.02 | 0.71 (0.29, 0.91) | 0.01 | 0.06 | 0.68 (0.23, 0.90) |
| JAC Axis 2 | -0.13 | -0.04 | 0.86 (0.65, 0.96) | -0.13 | -0.09 | 0.89 (0.69, 0.97) | -0.13 | 0.02 | 0.78 (0.43, 0.93) |
|  |  |  |  |  |  |  |  |  |  |
|  | **FIT vs. FOBT** |  |  | **FIT vs. RNAlater** |  |  | **FOBT vs. RNAlater** |  |  |
| **Microbiome metric** | **FIT Mean** | **FOBT Mean** | **ICC (95% CI)** | **FIT Mean** | **RNAlater Mean** | **ICC (95% CI)** | **FOBT Mean** | **RNAlater Mean** | **ICC (95% CI)** |
| Actinobacteria^a^ | 0.014 | 0.029 | 0.55 (0.02, 0.85) | 0.014 | 0.029 | 0.73 (0.34, 0.90) | 0.029 | 0.029 | 0.87 (0.64, 0.96) |
| Bacteroidetes^a^ | 0.393 | 0.389 | 0.63 (0.14, 0.88) | 0.393 | 0.245 | 0.85 (0.58, 0.95) | 0.389 | 0.245 | 0.84 (0.56, 0.95) |
| Firmicutes^a^ | 0.530 | 0.508 | 0.65 (0.16, 0.89) | 0.530 | 0.670 | 0.91 (0.74, 0.97) | 0.508 | 0.670 | 0.79 (0.46, 0.94) |
| Observed species | 561 | 552 | 0.88 (0.67, 0.97) | 561 | 535 | 0.85 (0.57, 0.95) | 552 | 535 | 0.98 (0.93, 0.99) |
| Observed genes | 30,893 | 32,443 | 0.64 (0.15, 0.88) | 30,893 | 28,801 | 0.73 (0.35, 0.91) | 32,443 | 28,801 | 0.95 (0.84, 0.98) |
| Shannon index for species | 8.661 | 8.720 | 0.83 (0.53, 0.95) | 8.661 | 8.523 | 0.72 (0.32, 0.90) | 8.720 | 8.523 | 0.88 (0.65, 0.96) |
| Shannon index for genes | 2.88 | 2.99 | 0.69 (0.24, 0.90) | 2.88 | 2.73 | 0.64 (0.20, 0.87) | 2.99 | 2.73 | 0.88 (0.67, 0.97) |
| BC Axis 1 | -0.01 | 0.03 | 0.92 (0.78, 0.98) | -0.01 | -0.06 | 0.95 (0.84, 0.98) | 0.03 | -0.06 | 0.92 (0.78, 0.98) |
| BC Axis 2 | -0.05 | -0.08 | 0.80 (0.47, 0.94) | -0.05 | 0.01 | 0.89 (0.67, 0.96) | -0.08 | 0.01 | 0.82 (0.51, 0.95) |
| JAC Axis 1 | 0.01 | -0.02 | 0.92 (0.78, 0.98) | 0.01 | 0.06 | 0.93 (0.79, 0.98) | -0.02 | 0.06 | 0.91 (0.75, 0.97) |
| JAC Axis 2 | -0.04 | -0.09 | 0.81 (0.50, 0.94) | -0.04 | 0.02 | 0.89 (0.66, 0.96) | -0.09 | 0.02 | 0.83 (0.54, 0.95) |

^a^ Phylum relative abundances were square root transformed prior to calculating ICCs
